# Copper Recycling Flow Model for the United States Economy: Impact of Scrap Quality on Potential Energy Benefit

**DOI:** 10.1021/acs.est.0c08227

**Published:** 2021-03-30

**Authors:** Tong Wang, Peter Berrill, Julie B. Zimmerman, Edgar G. Hertwich

**Affiliations:** † Department of Chemical and Environmental Engineering, Yale University, New Haven, Connecticut 06520, United States; ‡ Center for Industrial Ecology, Yale University, New Haven, Connecticut 06520, United States; § Yale School of the Environment, Yale University, New Haven, Connecticut 06520, United States; ∥ Industrial Ecology Programme, Department of Energy and Process Engineering, Norwegian University of Science and Technology (NTNU), 7495 Trondheim, Norway

## Abstract

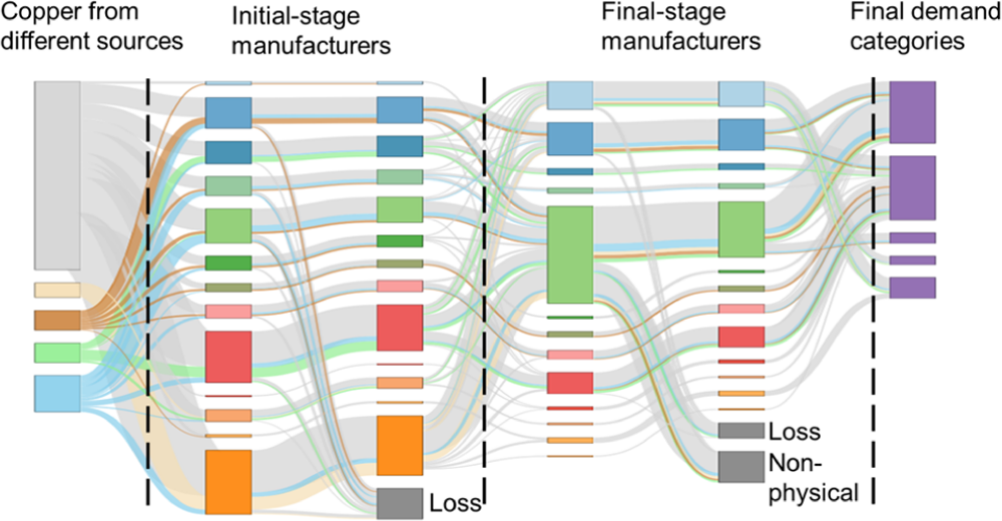

Is recycling a means for meeting the increasing copper demand in the face of declining ore grades? To date, research to address this question has generally focused on the quantity, not the quality of copper scrap. Here, the waste input–output impact assessment (WIO-IA) model integrates information on United States (US) economy-wide material flow, various recycling indicators, and the impact of material production from diverse sources to represent the quantity and quality of copper flows throughout the lifecycle. This approach enables assessment of recycling performance against environmental impact indicators. If all potentially recyclable copper scrap was recycled, energy consumption associated with copper production would decrease by 15% with alloy scrap as the largest contributor. Further energy benefits from increased recycling are limited by the lower quality of the scrap yet to be recycled. Improving the yield ratio of final products and the grade of diverse consumer product scrap could help increase copper circularity and decrease energy consumption. Policy makers should address the importance of a portfolio of material efficiency strategies like improved utilization of copper products and lifetime extension in addition to encouraging the demand for recycled copper.

## 1 Introduction

Copper demand has more than doubled in the past 40 years,^1^ a trend that is estimated to continue in the forthcoming decades.^2,3^ The most important end uses of copper globally in 2017 were equipment manufacturing (31%), building construction (28%), and infrastructure (16%).^4^ While low-carbon technologies are more environmentally friendly in terms of lower emissions causing climate change, freshwater ecotoxicity, and so forth, they tend to require more copper than the current energy technologies.^5−7^ In contrast to other major industrial metals like steel,^8^ projections from both technology-based^3,9,10^ and econometric^3,11^ analyses indicate that in-use copper stocks will not reach saturation in the coming decades. Although aluminum, steel, plastics, graphene, and optical fiber can substitute for copper in various applications, they have relatively poor performance in the end-use categories that together account for more than 70% of copper use.^12^ Production-related land use (like mineral extraction site), energy and water consumption, human toxicity, and greenhouse gas (GHG) emissions present external costs associated with copper supply.^13,14^ Mining and mineral processing stages account for 60–90% of energy requirement for primary copper production depending on the ore grade and are significant in generating various environmental burdens.^2,13,15,16^ GHG emissions from copper products in 2015 were 180 Mt, 0.36% of global emissions, up from 105 Mt in 1995.^15^ 71–93% of GHG emissions from copper production are contributed by electricity supply,^16^ making energy use a significant contributor to the environmental footprint of copper products.

Although researchers disagree on the question of whether the world will experience growing copper scarcity in the following decades while continuing the trend of increased copper use,^13,17−20^ collectively, researchers have emphasized the importance of copper recycling when considering long-run cleaner copper supply and environmental impact alleviation.^2,3^ While Elshkaki et al.^2^ and Schipper et al.^3^ suggest that unless a high recycling rate is achieved, copper resources will be exhausted in the next decades, others argue that new discoveries and improvements in mining technologies will increase copper resources, as they have done for oil and gas.^18,19^ Meanwhile, Northey et al.^13^ and Mudd et al.^17^ suggested that the identified copper resources seem to be sufficient to meet the growing global demands for the next decades, albeit with some ore grade decline. Despite the debate on the sufficiency of copper resources, copper recycling, especially of high-grade copper scrap, requires significantly less energy, land, and water than primary production.^2,21^

There is a substantial potential to increase the recycling rate of copper. Although about 95% of used copper is “potentially recyclable,”^22^ 26–82% of end-of-life copper scrap is recycled depending on the use category, location and period^23−27^ with a 10-year average of 40% at the global level.^25^ Similarly, copper scrap constitutes around 20–50%^23−26,28^ of the copper resource used in the production of new copper with a 10-year average of 32% at the global level.^25^ Following the material flow analysis (MFA) literature,^22,23^ the end-of-life recycling rate (EoL-RR) is defined as the percentage of copper in discards that is actually recycled. Inefficiencies in collection, separation, and processing are the main reasons for a low EoL-RR.^27^ The recycling input rate (RIR) is defined as the proportion of metal that is produced from both new (production waste) and old (postconsumer) scrap, while EoL-RIR is the proportion only from old scrap (see the Supporting Information). Given the long lifetime and growing production of copper-containing products and infrastructure and imperfect recycling efficiency, the EoL-RIR is lower than the EoL-RR,^29^ which means that only part of the copper demand could be met by secondary production even at a high EoL-RR; a circularity gap exists.^30^ Dong et al.^10^ modeled copper demand using a bottom-up dynamic stock and flow analysis and suggested that only about 50% of 2050 copper demand in China could be covered by secondary copper in an ideally high EoL-RR scenario.

The demand for complex end-use products drives the generation of complex and low copper-content scrap. Electronic products, for example, use dozens of metals in production, with copper being most important in terms of mass.^31^ The electronic scrap contains only about 3–30% copper due to its complexity; thus, recycling requires more metallurgical processing. It is defined as low-grade copper scrap.^32,33^ New scrap is generated during the manufacturing process, and most of it is directly melted to produce semifinished goods (semis as the products of the first processing stage from refined copper including copper wire, bars, rods, etc. are called) due to its high quality.^26^ Based on the copper content, there are mainly four types of EoL copper scrap:^34,35^ three types of refined copper scrap, no. 1 scrap (>99% Cu content), no. 2 scrap (88–99% Cu), and low-grade copper-bearing scrap (10–88%), and Cu alloy scrap (around 60% Cu). Low grade and complex copper scrap are anticipated to cause more energy consumption and environmental impact. For example, recycling of low-grade copper scrap for semis production was estimated to consume more than double the energy compared with that of no. 2 scrap.^35^

To make better use of recycling as a strategy to sustain copper supply, it is crucial to understand the economy-wide flows of copper from different sources including scrap of different qualities and the associated energy consumption. MFA quantifies material flows in and among economic sectors.^36,37^ Process-based MFA quantifies copper flows by looking at flows and stocks at different life stages and the copper use in aggregated end-use sectors like buildings and transportation.^10,24,26,38^ More comprehensive production processes^36^ and end-use sectors^10,26^ have been included in recent years in metal MFAs. Input–output (IO)-based MFA is able to explore copper use in all economic sectors as well as intersectoral flows.^39,40^ However, past studies did not trace the concentration of copper or the quality of potentially recovered scrap. In order to address the waste sectors, researchers have combined MFA with IO tables to develop the waste input–output material flow analysis (WIO-MFA) method^41,42^ that was applied in this paper. The WIO-MFA method can be used to derive the composition of products while avoiding double counting. Due to the limited product-level detail of IO tables, the types of copper scrap in the waste stream have not yet been detailed as has been presented here. Moreover, few IO-based MFAs have explored the detailed manufacturing process in terms of first-stage manufacturers (e.g., of metal rods, wire, and castings), component manufacturers (e.g., of insulated cables, motors, and valves), and final-stage manufacturers (e.g., of buildings and automobiles)^43^ as was done in this study. Previous research on estimating energy savings by recycling have integrated energy consumption for both primary and secondary copper production with MFA.^27,44^ While most research concentrated on the overall potential and used aggregated energy information of copper recycling without considering specific scrap types, Ciacci et al.^45^ have improved the estimation through differentiating energy consumption for secondary production between direct melting and smelting. Nevertheless, the effects of detailed scrap types covering all copper content ranges like low-grade copper scrap have not been explicitly addressed. A framework that offers comprehensive assessments of economy-wide material flows from sources of different quality and the associated environmental impact is still lacking.

This paper aims to explicitly map the copper flows from different sources across the United States (US) economy and quantify the energy savings of recycling various copper scrap qualities by economic sector. Its two main goals are (1) to map how copper from different sources enters the US economy and flows among sectors to be embedded in the final demand and (2) to assess the energy consumption in each economic sector due to the copper composition as a result of various processing steps and explore different recycling scenarios at an economic level. We extended the existing WIO-MFA to a model called waste input–output impact assessment (WIO-IA) and integrated the information of RIR, EoL-RR, scrap types, and the corresponding energy consumption of copper semis production. This paper is an economy-wide analysis on the role of recycling copper scrap of different qualities in forming the copper use patterns in economic sectors and in decreasing the associated energy consumption.

## 2 Methods

### 2.1 WIO-IA Model Part I—Characterizing Copper Requirements

**Figure 1:**
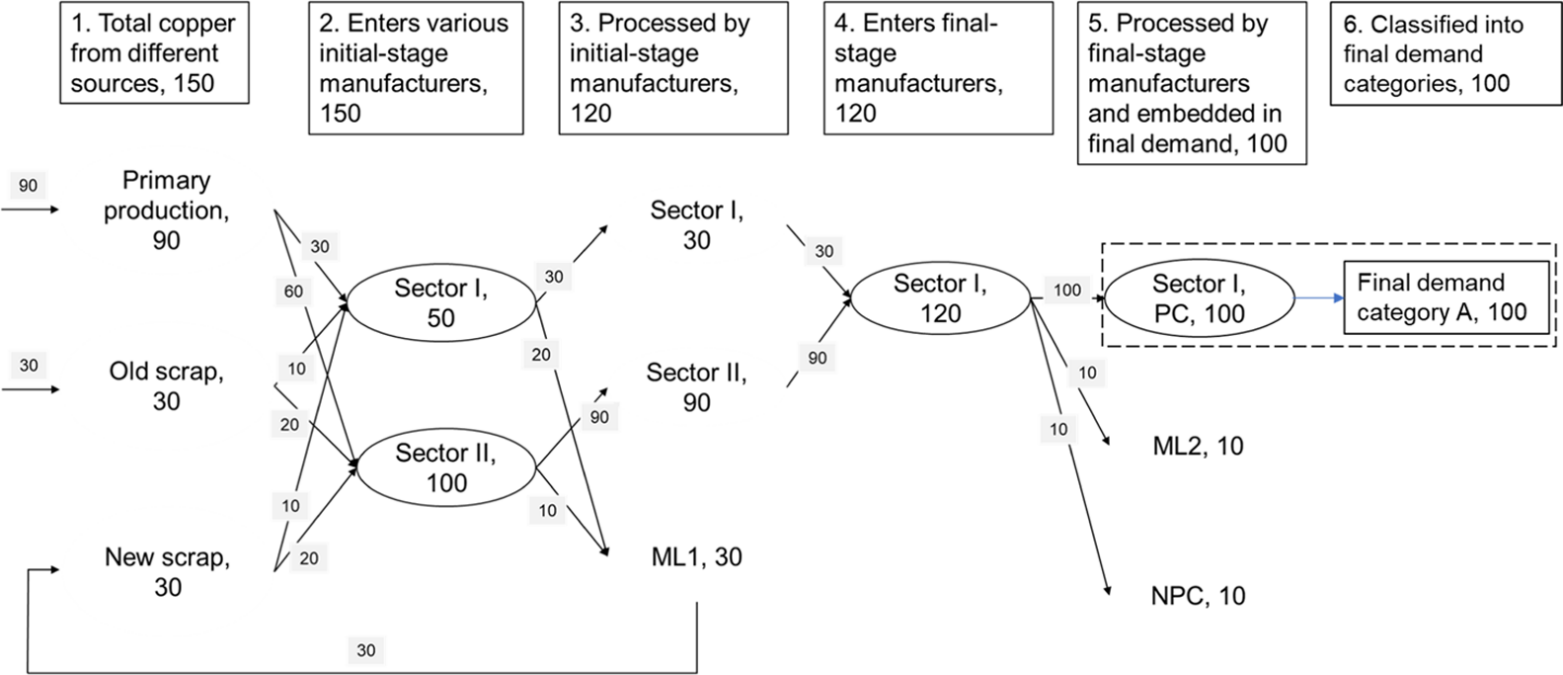
Simplified framework exemplifying copper flow to deliver 100 t of copper content in sector I in an economy. PC represents physical content. ML1 represents the manufacturing loss as new scrap from initial-stage manufacturers. ML2 represents the manufacturing loss during the processing by final-stage manufacturers. The dashed line area shows different classifications rather than actual flows. Each of the flow arrow after step 2 could also be differentiated by copper sources which is not shown in this simplified figure but is identified in the results.

The simplified framework for characterizing copper requirements is shown in Figure 1. When copper semis enter the economy from different sources, they support the final demand in one of the following four forms: physical content (PC), manufacturing loss (ML), service, and product produced with copper but not containing copper. The latter two can be aggregated as nonphysical copper (NPC) requirements that are required to satisfy the final demand in the form of products or services that require copper to produce but do not contain copper. This paper aims to explicitly quantify these components, PC, ML, and NPC, that account for the total amount of copper entering the economy. The embedded copper in economic sectors is impacted by the direct copper input into the economy and the various processing steps. We denoted the sectors whose embedded copper is mainly from direct copper input as initial-stage manufacturers which include first-stage manufacturers and component manufacturers following the terminology in Graedel et al.^43^ The final-stage manufacturers incorporated the copper from initial-stage manufacturers. For example, a crucial part of copper embedded in residential buildings in final demand is from initial-stage manufacturers in the form of cables.

In this paper, we denoted the matrix that represents the proportions of direct copper input from different sources to the US economic sectors as the proportion matrix (eq 1). Two assumptions were made in this paper to derive the proportion matrix: the direct secondary copper input to each sector is from the copper scrap type that is generated by this sector and the portion of copper from the EoL scrap in the direct copper input is proportional to EoL-RR. The first part of the assumption is similar to the idea of the “pseudoclosed loop scrap allocation” in which products only use their own scrap.^46^ One type of EoL scrap was assigned to each sector according to literature, and the portion of copper from this type of EoL scrap in the proportion matrix equals to EoL-RIR. The second assumption is meant to address the impact of increased EoL-RR on the proportion matrix under a static scrap processing efficiency (including collection, dismantling, sorting, separation, smelting, converting, and refining process) and copper demand. EoL-RIR is the statistical information on the EoL scrap availability to meet the total copper usage.^26,47^ For a specific sector, a higher EoL-RR means more copper is available to be used in this sector’s copper input, and we derived EoL-RIR by assuming that the coefficients of EoL-RR/EoL-RIR for all sectors were the same as the economy-wide EoL-RR/EoL-RIR. The proportion of new scrap in the direct input was set to be the loss of copper in initial-stage manufacturing. Based on the proportion matrix, we could disaggregate the matrix that represents the direct input of copper per unit of product into copper from different sources. In total, there are six types of copper sources: primary production, new scrap, and four EoL scrap types including no. 1 scrap, no. 2 scrap, low-grade copper-bearing scrap, and Cu alloy scrap (see detailed definition in Introduction). The element (i,j) of the proportion matrix, *P*_i,j_, indicates the proportion of the direct copper material input from source i to sector j. The sum of all six columns for each row equals 1.
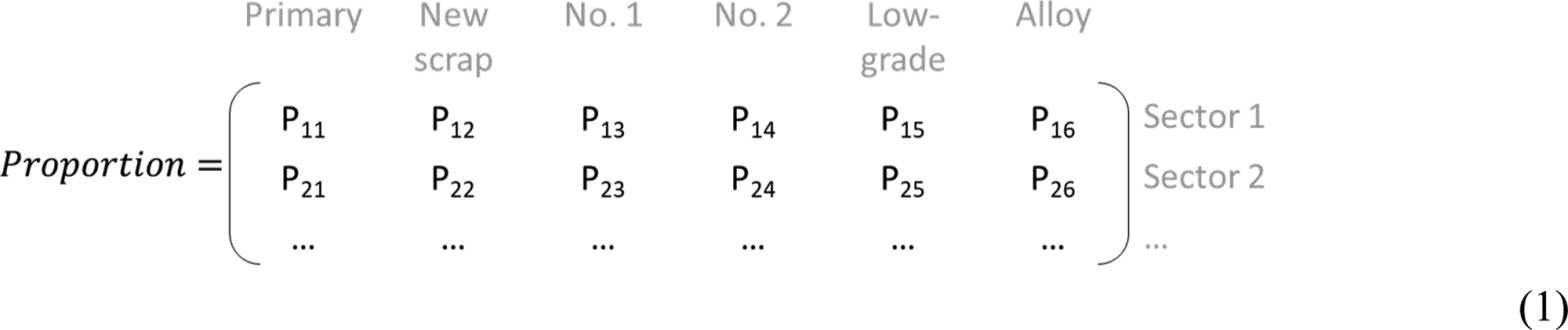
1

The technical coefficient matrix *A* and the final demand matrix *y* from the most recent US IO table, the 2012 USEEIO table,^48,49^ was used in this paper. Based on the degree of fabrication, economic sectors are categorized as “resources,” “materials,” or “products” following the WIO-MFA procedure.^41,42^

The total copper requirements from different sources for the final demand, CUs_total_, is calculated as
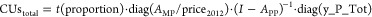
2where *t*(proportion) is the transpose of proportion matrix; *A*_MP_ is the direct monetary input intensity from “materials” to “products” sectors; *A*_PP_ is intersectoral monetary intensity flows among the “products” sectors; price_2012_ is the copper semis price in 2012; y_P_Tot is the vector representing the total final demand of “products” sectors; and diag(·) represents the diagonalization of this vector. See the Supporting Information 1–2 for detail.

### 2.2 WIO-IA Model Part II—Assessing Impact

The second component of the WIO-IA model is to integrate impact information in the model. Total energy demand required to satisfy the demand for semifinished copper by economic sector, energy, is calculated as
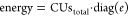
3where diag(*e*) is the diagonal form of *e*—the vector representing life cycle cumulative primary energy demand per unit of copper material from various sources.

For copper primary production, the energy requirement covers all upstream energy consumption including mining, mineral processing, smelting, refining, and semis production. Data from both literature and own calculation using Ecoinvent 3.6^50^ were considered. An uncertainty range induced by various ore grades and technology utilized was provided and reflected in the results as error bars for primary copper production. For secondary production, energy requirements were differentiated among all five types of copper scrap. Detailed energy information is provided in Supporting Information 1–8.

### 2.3 Scenario Analysis

Higher collection rates and better sorting of copper scrap can potentially improve recycling performance in terms of increased EoL-RR, EoL-RIR, and reduced energy consumption. In addition to the current situation as the base case scenario, we carried out another two scenarios of increasing EoL-RR for various economic sectors to identify the potential to reduce energy consumption: scenario 1, increasing EoL-RR of all end-use categories by 10 percentage points; scenario 2, increasing EoL-RR of each end-use category to recycle all potentially recyclable^22^ copper. In scenario 2, we derived EoL-RR by estimating the proportion of “potentially recyclable”^22^ copper (excluding in-use dissipated loss, currently unrecyclable portion, and other unspecified parts) in EoL copper scrap generated. See details in Supporting Information 1–10. We did not explicitly model in-use loss which is only around 1% in all copper-containing product globally^22^ but adopted the EoL-RR information (or “potentially recyclable”^22^ concept in scenario 2) from literature which has already excluded the in-use loss from recycled (or recyclable) portion. Changing EoL-RR will change the proportion matrix and thus change the embedded copper composition and the energy input of per unit sector output. Moreover, in order to assess the influence of factors other than recycling on copper demand and associated energy use, we conducted scenario 3 to reflect technical coefficient and behavioral change by referring to the US vehicle situation in 2019. In scenario 3, wire and direct copper semis input into per unit output of motor electrical and electronic equipment and motor transmission and power train parts was increased by 0.4%, and the final demand for automobiles, light trucks, and heavy trucks was about 65, 170, and 152% of those in 2012, respectively.^28,51,52^ See the detailed explanation for scenario 3 in the Supporting Information 1–11.

The detailed steps to calculate for each stage in Figure 1 and the data used are described in the Supporting Information.

## 3 Results

### 3.1 Economy-Wide Copper Flow from Various Sources to Final Demand

**Figure 2:**
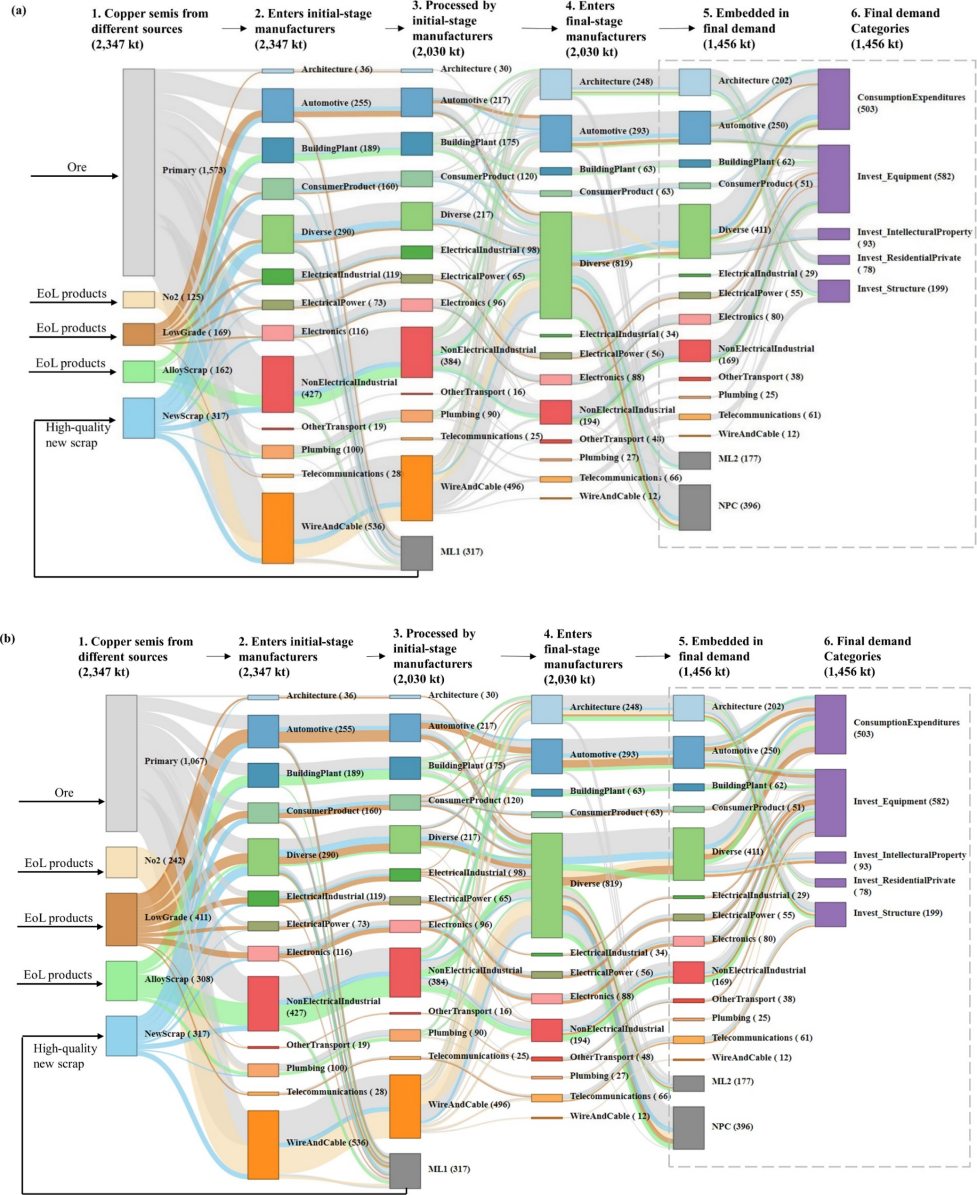
Economy-wide copper flow from various sources to final demand in the 2012 US economy. (a) represents the base case scenario, and flows less than 6 kt were omitted from this figure for easy reading. (b) represents scenario 2 in which the EoL-RR of each sector was derived from its potentially recyclable rate,^22^ and flows less than 5 kt were omitted from this figure for easy reading. PC represents physical content. ML1 represents the manufacturing loss as new scrap from initial-stage manufacturers. ML2 represents the manufacturing loss during the processing by final-stage manufacturers. The dashed line area shows different classifications rather than actual flows.

This study mapped the copper flow in the US economy from semis supply to the final demand and identified the key sectors. According to the WIO-IA model, the total copper requirement in the 2012 US economy was estimated to be 2,347 kt (thousand metric tons) with an RIR of 33%. The estimation is comparable to the actual 2012 US copper supply which was reported as 2464 kt (including 637 kt of net import) with a RIR of 33%.^53^Figure 2a shows the detailed copper flows within the 2012 US economy. It indicates that there are usually several processing steps before copper enters the product where it fulfils its ultimate function, such as conducting electricity or heat. Copper semis were used mainly by initial-stage manufacturers like wire and cables and some nonelectrical industrial sectors like valves and fittings. After accounting for the processing steps, a significant amount of copper ended up in other categories like architecture, automotive, and diverse uses (including services, ammunition, clothing, etc; see the Supporting Information for details). The major contributors to the embedded copper in the diverse category were the sectors providing services. About 19, 9, and 7% of the total copper embedded in the diverse category were in the “scientific research and development,” “nonsatellite wireless telecommunications carriers,” and “federal government (defense)” sectors, respectively. More than half of copper reaching final demand was for investment in equipment, structures, and so forth. Figure 2b represents the situation in scenario 2 where EoL-RRs were estimated from the potentially recyclable rates,^22^ and EoL-RIRs increased accordingly. Although the copper demand among sectors/categories was the same from stage 2, the copper sources changed remarkably. The copper from low-grade copper scrap was more than doubled, and EoL-RIR would increase from 20 to 41%. The copper use patterns changed for each sector in terms of copper sources, and energy demand would change as in the later sections.

About 38% of the total copper used in the 2012 US economy did not reach the final demand according to this model. It was either lost during the manufacturing process or was used in an intermediate use that does not have physical copper output to final demand. The manufacturing loss in step 5 (ML2) was not directly recycled as high-quality new scrap as it represents the loss during final-stage manufacturing where products become more complex and the scrap quality varies. The manufacturing loss rates during initial-stage (ML1) and final-stage (ML2) manufacturing were estimated to be 13.5% (317 kt/2347 kt) and 8.7% (177 kt/2030 kt), respectively, which are comparable to the global-level estimation of an average 16% input rate of new scrap during 2000–2010^26^ and a 7.6% loss rate during the final-stage manufacturing of copper semis for end-use in 2012.^54^ The diverse category had a comparatively large portion become ML or NPC. For example, pesticides that contain copper were used during agricultural production, but the products of the agricultural sectors do not contain copper from pesticides. Thus, the used copper in pesticides for agricultural production was considered as NPC.

**Figure 3:**
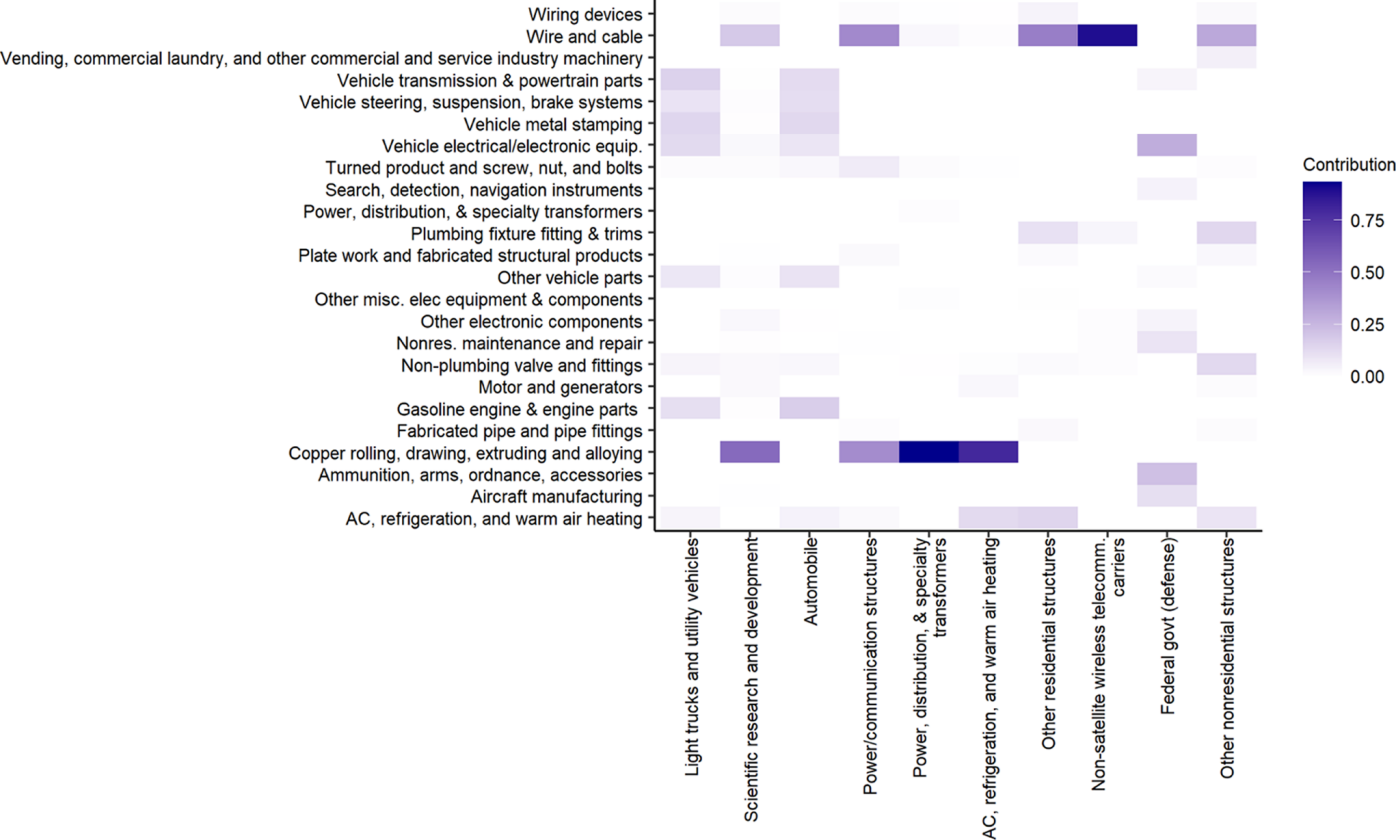
Contributions of various sectors to the top 10 sectors in terms of total embedded copper in the 2012 US economy. *X*-axis represents the top 10 final demand sectors in decreasing order. *Y*-axis shows the major contributors of each top sector, and the color in each cell shows the portion contributed by a certain sector for the corresponding top demand sector as illustrated in the legend.

As shown in Figure 3, copper in products consumed by final demand in the 2012 US economy was added primarily in the form of wires and cables, motor parts, air conditioning, plumbing fixtures, valves, and fittings. Automotive equipment, power utility, communication structures, and building construction were among the top final demand sectors absorbing copper. For the automotive equipment, embedded copper was mainly from motor parts. Embedded copper in the power and communication sectors consists of copper from direct input and wire and cables. Wires and cables, plumbing, and thermal comfort equipment contributed crucial portions to the embedded copper in buildings. It is noteworthy that the “scientific research and development” sector had noticeable copper inputs from a variety of sectors, although it did not have physical copper output to other sectors. About 54% of the embedded copper in this sector was from direct copper semis input, followed by about 19% from wires and cables. For each final demand category, top final demand sectors and their contributors were analyzed and are shown as heatmaps in the Supporting Information.

### 3.2 Embedded Copper Use in Typical Sectors and Related Energy Consumption

Specific copper (embedded copper per monetary unit of sector) for all economic sectors in the 2012 US economy was estimated in this model and is provided in the Supporting Information. The plausibility of the estimated specific copper was checked and is shown in Table S8 before further analysis.

**Figure 4:**
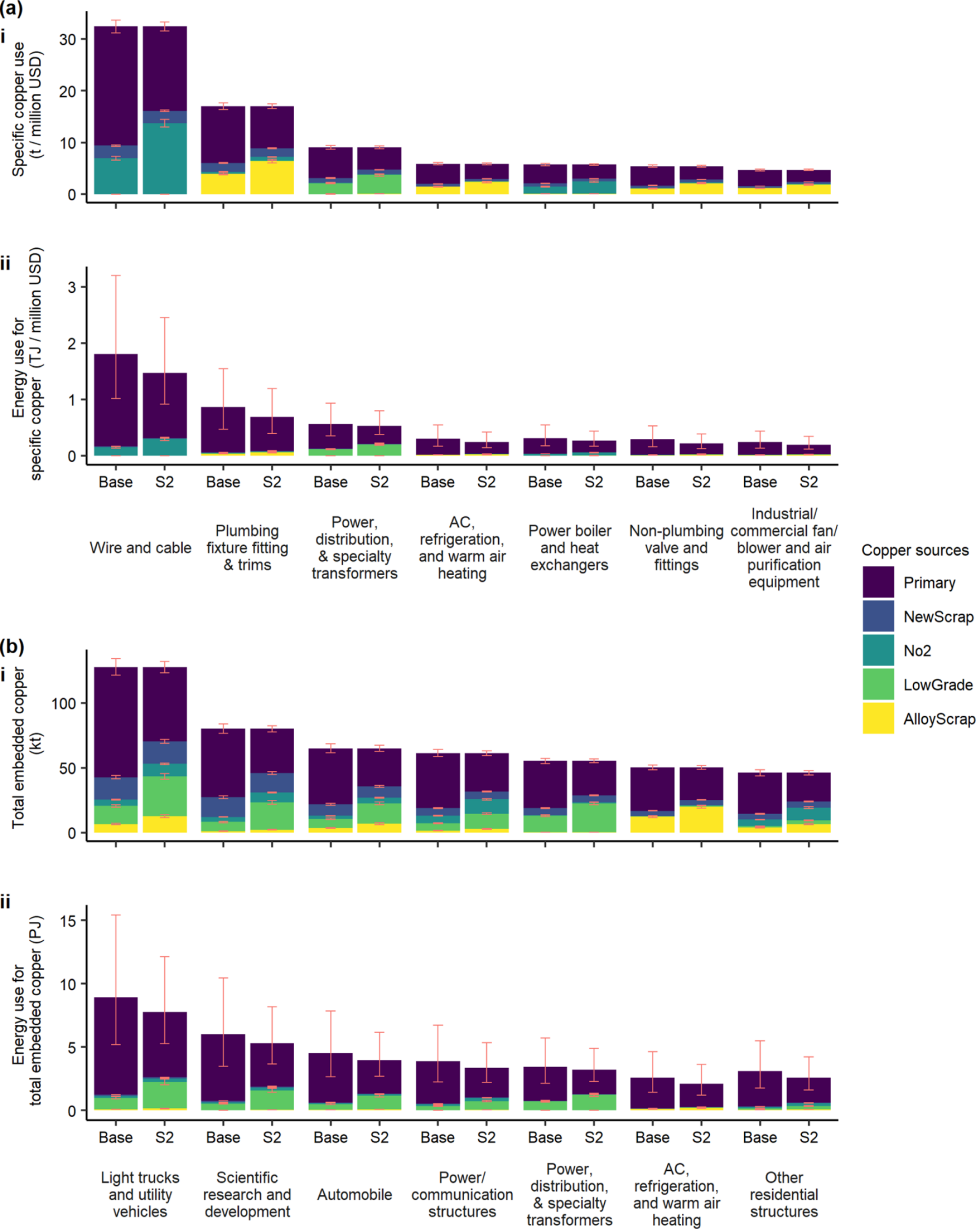
Embedded copper use in important sectors and related energy consumption. (a)-i represents top seven specific copper use sectors under base case scenario (base) and their specific copper use under scenario 2 (S2) in the 2012 US economy and (a)-ii represents the associated energy use. (b)-i represents top seven total embedded copper use sectors under base case scenario (base) and their total embedded copper use under scenario 2 (S2) in the 2012 US economy and (b)-ii represents the associated energy use. Error bars in (a)-i and (b)-i represent the uncertainty in fabrication efficiency (see the Supporting Information). Error bars in (a)-ii and (b)-ii represent the uncertainty in the energy consumption for per unit copper production from different sources (see the Supporting Information).

The “Wire and cable” sector had the highest specific copper use (Figure 4a-i). As an important initial-stage manufacturer, most of its copper was from direct copper input and had a relatively simple composition of copper from primary sources, new scrap and no. 2 scrap. It also ranked first when considering the energy consumption for specific copper use (Figure 4a-ii). The primary copper use provides the largest contribution to energy consumption, with the magnitude depending on ore grades and technology utilized. The top embedded copper sectors had a relatively complex copper composition as a result of copper input from various initial-stage manufacturers. “Light trucks and utility vehicles” ranked first for both total copper and energy use (Figure 4b-i,ii). With primary copper being the most significant, it consisted of five types of copper sources. In scenario 2, the copper use patterns changed significantly for each of the top sectors in terms of more copper from EoL scrap. The portion of energy consumption associated with copper produced from EoL scrap increased, but the overall energy consumption of copper from all sources decreased. With EoL-RIR increasing, the order of energy consumption associated with specific copper use changed due to scrap quality. For example, the rank of energy use for specific copper use in the air conditioning sector became lower in scenario 2 than in the base case as more alloy scrap was recycled. It implied the importance of considering scrap quality in addition to quantity when considering energy reduction by recycling.

### 3.3 Total Embedded Copper and Associated Energy Consumption under Scenarios

Total energy consumption for copper production in the US in 2012 was estimated to be 117 PJ (as the representative value) in this model, and the values for China and the world in 2010 were about 536 and 2000 PJ in the studies by Dong et al.^55^ and Elshkaki et al.,^2^ respectively. Energy consumption per unit of copper is within the range of values reported for other regions, as compared in Supporting Information 1–9.

**Figure 5:**
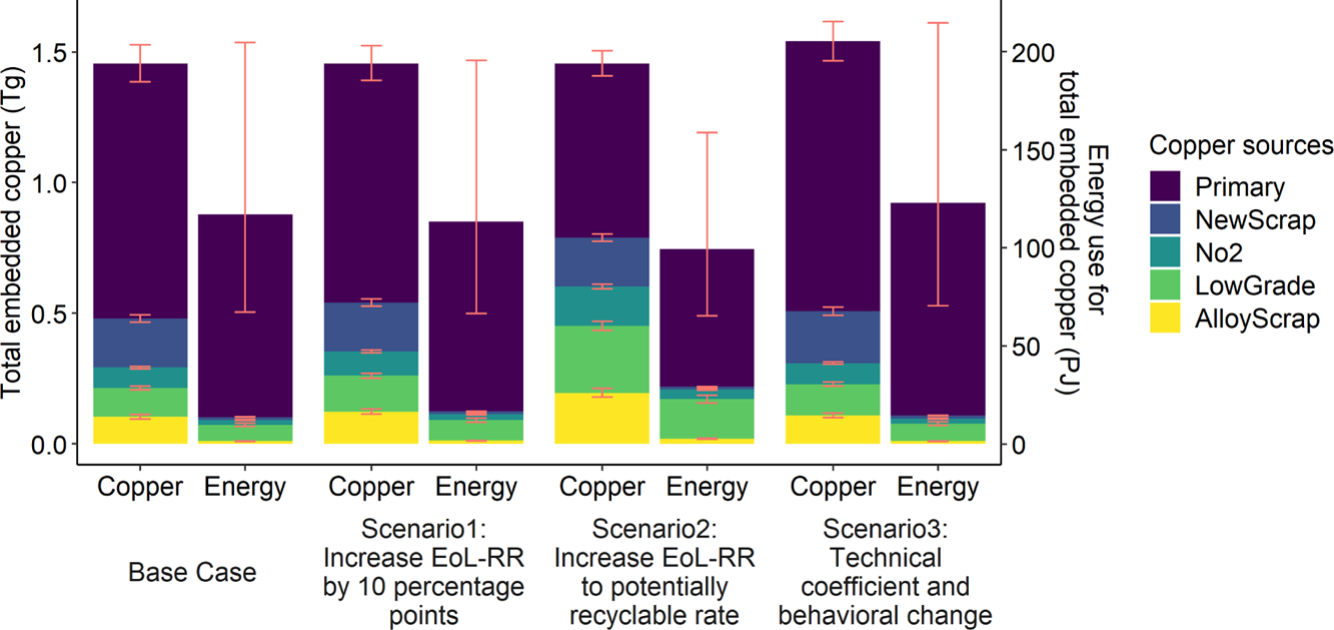
Comparison among scenarios for total embedded copper use in final consumption (left *y* axis) in the 2012 US economy and associated energy consumption (right *y* axis). Error bars for copper represent the uncertainty in fabrication efficiency (see Supporting Information). Error bars for energy represent the uncertainty in the energy consumption for per unit copper production from different sources (see the Supporting Information).

Both scenarios 1 and 2 achieved a reduction of total energy use by increasing the portion of copper from recycling (Figure 5). Although embedded copper in base case and scenarios 1 and 2 were the same, the sourcing of copper shifted substantially toward EoL scrap as EoL-RR increased. Compared with base case, the embedded copper from EoL scrap increased from 20 to 24% and 41% in scenarios 1 and 2, respectively. Despite the wide uncertainty range, primary copper production dominated the total energy consumption under all scenarios which implied the benefit of recycling. When increasing EoL-RR by 10 percentage points, total energy consumption associated with copper use decreased by 3% compared with base case. EoL-RR was about double in scenario 2 in which all potentially recyclable copper was recycled. The energy saving in scenario 2 was 15% in which 5, 3, and 7% were contributed by the increasing of EoL-RR in the no. 2 scrap, low-grade scrap, and alloy scrap generating sectors, respectively. When the EoL-RIR increased from current 20 to 41% in scenario 2, marginal energy saving decreased as more low-grade scrap was recycled (Figure S16). In scenario 3 where vehicles had higher per unit copper input and people in the US bought more trucks, copper requirements increased by about 6% compared with the base case. The associated energy use increased by about 5%. The less-than-proportional increase in energy use is because the average EoL-RR for economic sectors to fulfill the requirements of vehicles was larger or their scrap quality was higher than for the average copper-containing products according to the model. The additional copper use per unit of vehicle could be induced by more production of electric vehicles.^56,57^ Electrification of vehicle drive trains can reduce GHG emissions but requires more copper material.^5,57,58^ The results of scenarios 3 also implied the significant impact of behavioral change on copper demand.

## 4 Discussion

The WIO-IA model presented here integrated the information of economy-wide copper flow in the US from sources of different quality among various life stages, diverse recycling indicators, and energy consumption, and offered a platform to assess the performance of the current recycling system and different scenarios. It has advantages in providing a holistic economy-wide assessment of the copper flows from different sources to final demand in the economy, helping prioritize the sectors that have significant copper use for future analysis and identifying energy saving potentials. Overall, there is a substantial potential to increase the recycling of copper by recovering more copper-containing scrap and recycling it more efficiently, that is, with a higher yield for current products with lower EoL-RR like consumer and electronic products,^25,26^ but the energy savings are not as high as one might expect because more of this scrap is lower grade and hence requires more energy to be recycled than the high-grade scrap that dominates current recycling.

Copper initial-stage manufacturers had relatively high specific copper use and less complex copper composition. For the final demand, there were more copper sources involved in the production process. One way to address the responsibility of recycling could be assigning more responsibility to those producers that have substantial demand for copper and generate low-grade scraps, for example, by requiring a minimum recycled copper content. A large portion of copper final demand was used as investment in products with long life spans. For example, the lifespan of infrastructure and transport was estimated to be approximately 50 years and 20 years, respectively,^49^ with the annual growth rates comparable to the gross domestic product growth rates.^3^

Improved material efficiency could reduce future copper demand. According to the model results, the largest manufacturing losses of copper in the US occur in the production of a wide range of complex products/services such as automobiles and those that contain only small amounts of copper. If the yields are increased by 5 percentage points, 7.4% more copper semis would be able reach the final demand in this model, which implies an opportunity to reduce copper requirements and energy use when fulfilling the same amount of final demand. Beyond reducing production loss, life extension of important products like residential buildings^60^ can reduce copper demand and associated energy use, although a case-by-case assessment of benefits and trade-offs may be required and copper may be unimportant as a cause of overall impacts. For example, manufacturers use a small-diameter copper tubing technique on air conditioners which have higher heat transfer coefficients and lower material costs.^61^ If the life of the conventional less energy efficient air conditioners was extended, the additional energy loss would outweigh the benefit of saving copper. Detailed trade-offs could be assessed based on a dynamic stock and flow model for future air conditioner demand, new technologies, and life cycle assessment for both old and new technologies. Technological innovation, like modular design of consumer products^62^ and remanufacturing of electronics and home appliances, and behavioral change, like more intensive use of automobiles, are among the desirable material efficiency choices.^60,63^

Substitutes could potentially reduce copper use but raise two tradeoffs that require vigilant consideration. The first relates to the performance of substitutes. For example, substitutes like aluminum function well in architecture^12^ as ornamental metal products, but the performance of substitutes in a significant portion of the end-use categories, like electrical, electronic, and transport use,^12^ is inferior. The other type is about the environmental impact in the production process of the substitutes. For example, copper has lower cumulative energy demand and GHG emissions than aluminum but a higher human toxicity, freshwater eutrophication, and terrestrial acidification impact when comparing on a per kilogram basis.^14^ Overall, substitutes reducing the scarcity and cost of original material might raise the energy consumption and environmental impact during the production or in-use phase. Comprehensive circularity metrics, which quantify and compare the circularity between a material and its substitutes by considering economic, social, and environmental aspects in addition to material consumption, could inform decision makers of the tradeoffs at systems level to avoid burden shifting.^44,45,64−68^

Of all copper used in society, it is very difficult or even impossible to identify the highly uncertain input of scrap types and quantities into different economic sectors as the actions of recyclers were not explicitly studied.^46^ We adopted the “pseudoclosed loop scrap allocation” as it offers a clear accountability potentially informing extended producer responsibility. The second assumption in the method implies stable scrap recycling efficiency and demand in economy. Our judgment is that this assumption is reasonable for a mature economy like the US but may not be so for emerging economies like China with high growth rate of sectors like infrastructure and transport. Without the two assumptions, the overall amount of copper flow among economic sectors will not change, but the partitioning among various scrap types into each economic sector will be vaguer. The main conclusion that scrap quality limits the energy benefit from increased recycling does not depend on the two assumptions. Compared with these two assumptions, the wide range of primary copper energy consumption and the limited information on secondary copper energy consumption are larger sources of uncertainty. We also call for improved data on the scrap flows by quality to improve the accuracy of the result.

The WIO-IA model has inherent uncertainty shared with other IO-based methods—the price homogeneity assumption and the IO assembling process itself. Information on copper content, prices, and purchases of various products could help overcome this limitation. The pseudo scrap allocation assumption addresses the negative externality of each sector, but it could also limit the scrap utilization potential compared with a market-based allocation method,^46^ which falls outside of this study. As there are limited data on copper recycling for detailed scrap types, uncertainty exists in the energy consumption per unit of copper production from each copper scrap. Although it would not likely change the main results, more detailed research on current and innovative copper recycling technologies for different scrap types could provide significant insights in improvement potentials. Last but not least, new demands or substitutes may develop.

## 5 Implications

Technological and behavioral strategies should be combined to better use copper, supporting the transition to a sustainable future by providing sufficient copper sources and less environmental impact. Setting minimum recycled content standards and building a better recycling infrastructure can drive technological improvement to improve the EoL scrap collection rate and processing rate. Increasing the service efficiency of copper-containing products, hence requiring fewer products to deliver a service, or choosing products with a lower copper content to provide a service, like automobiles rather than light trucks, has the potential to curb copper demand. Scrap upgrading technologies and lifetime extension should be closely scrutinized before implementation as additional energy cost might exceed the benefit of copper saving. An important source of copper, waste electrical and electronic equipment (WEEE), has been exported from developed countries to developing countries and managed by informal recyclers, which have lower yields and higher environmental and health impacts^69−72^ and could be managed more efficiently and effectively through policy reforms. Extended producer responsibility policies on WEEE^73,74^ and end-of-life vehicles^74,75^ that assign more responsibility on producers and importers showed potential on recycling innovation and awareness improvement.

While this paper used energy use as an indicator of impact due to its intrinsic importance and wide usage, other impacts such as GHG emissions or toxic emissions could be adopted in the model. This model could inform policy making by revealing the major material-use sectors among life stages, identifying opportunities to improve recycling performance, and assessing a portfolio of strategies.

## Supporting Information Available

Detailed description of equations, data sources, glossary, and other results (PDF)
2012 US IO matrices, other data used in the WIO-IA model, and selected results (XLSX)
