# Correction to “Copper Recycling Flow Model for the United States Economy: Impact of Scrap Quality on Potential Energy Benefit”

**DOI:** 10.1021/acs.est.3c02164

**Published:** 2023-05-02

**Authors:** Tong Wang, Peter Berrill, Julie B. Zimmerman, Edgar G. Hertwich


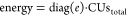
3

