# Supplementary material for: Copper Recycling Flow Model for the United States Economy: Impact of Scrap Quality on Potential Energy Benefit
Source: Environ Sci Technol. 2021 Mar 30;55(8):5485–95. doi: 10.1021/acs.est.0c08227 (PMC8154355; doi:10.1021/acs.est.0c08227)
Supplement: Supplementary file 1 — es0c08227_si_001.pdf [file es0c08227_si_001.pdf]

Supporting Information 1

Copper recycling flow model for the United States Economy: Impact of scrap quality on potential energy benefit

*Tong Wang[1,2], Peter Berrill[2,3], Julie B. Zimmerman[1,3], Edgar G. Hertwich[4, *]*

[1] Department of Chemical and Environmental Engineering, Yale University

[2] Center for Industrial Ecology, Yale University

[3] Yale School of the Environment, Yale University

[4] Industrial Ecology Programme, Department of Energy and Process Engineering, Norwegian University of Science and Technology (NTNU), 7495 Trondheim, Norway

*Corresponding Author: Edgar G. Hertwich: edgar.hertwich@ntnu.no

Number of pages: 33

Number of figures: 16

Number of tables: 8

APPENDIX S1-1. Glossary.

An easy understanding of copper recycling is that the copper-bearing product re-enters the production process when it comes to the end of life (EoL). However, there are plenty of concepts related to copper recycling, like copper scrap, recycling rates, etc., which might make the researchers and policy makers confused. The following definitions are relevant to this research.

Primary production[1]*:* Refined copper produced from ore, concentrate, or precipitate and copper cathodes from the solvent extraction-electrowinning process associated with dump, heap or roast-leach operations.

Old scrap[1] *(or postconsumer scrap)*[2]*:* Metal in products that have reached their end-of-life (EoL). Its recycling requires more effort, particularly when the metal is a small part of a complex products. It may be processed into refined copper ore used in the production of castings, mill products, chemicals, and other products.

New scrap[1]*:* Copper that is recovered from copper and copper alloy scrap generated during manufacturing (turnings, stampings, defective parts, etc.) and returned to smelters, fire refineries, brass mills, etc. for reprocessing. It may be directly melted into new products or processed into refined copper. It does not include home or runaround scrap generated within a plant and reprocessed at the same plant.

EOL-RR [2]: End-of-life recycling rates, the percentage of a metal in discards that is actually recycled.

RIR [3]: Recycling input rate. The RIR measures the proportion of metal and metal products that are produced from scrap and other metal-bearing low-grade residues (both old and new scrap).

EoL RIR [3]: End-of life recycling input rate. The EoL RIR measures the proportion of metal and metal products that are produced from EoL scrap and other metal-bearing low grade residues (only EoL scrap).

APPENDIX S1-2. WIO-IA model description

Although the 2012 USEEIO does not separately identify primary copper production, we acquired the primary copper production sector by disaggregating the nonferrous sector using the relations between primary copper production sector and nonferrous primary production sector in the 2007 USEEIO table[4,5]. Copper mining and refined copper production were chosen as “Resources”. Semi-finished copper which is represented by Sector “Copper rolling, drawing, extruding and alloying” with a North American Industry Classification System (NAICS) code of 331420, was chosen as “Materials”, and its’ direct input intensity to “Products” sectors is used as matrix A_{MP}. It includes rolling, drawing, or extruding shapes from purchased copper, alloying purchased copper and recovering copper and copper alloys from scraps [6], and acts as the semi-finished copper fabrication step. Copper use estimated in this research was compared with other data sources in the Results and Discussion section, suggesting the acceptability of the assumption of using Sector 331420 as “Material”. Other copper-using sectors were designated as “Products” and the intersectoral monetary intensity flows among the “Products” are used as matrix A_{PP}.

Overall, both A_{MP} and A_{PP}were split out from the A matrix to separate "Materials" and "Products".

To explore the copper direct input into the initial-stage manufacturers due to final demand (Step 1 – Step 2 in Figure 1):

$$CuDirInput = t(Proportion) \cdot diag(A_{MP}/Price_{2012}) \cdot diag((I - A_{PP})^{-1} \cdot y\_P\_Tot\ ) \qquad (1)$$

where the (i,j) element of $CuDirInput$ represents the direct input of copper semis from source i into sector j to meet the total final demand; $t(Proportion)$ is the transpose of Proportion matrix; $Price_{2012}$ is the copper semis price in 2012; y_P_Tot is the vector representing the total final demand of products. In this paper, we considered the total demand of copper happening inside the US by including the copper-containing product from abroad that were imported (domestic technology assumption) and excluding domestically produced copper-containing product that were exported.

To calculate the new scrap generation (Step 2 to Step 3 in Figure 1):

$$NewScrapGen = diag(colSums(CuDirInput)) \cdot Proportion[,2] \qquad (2)$$

where $colSums(CuDirInput)$ represents the column sum of $CuDirInput$ matrix; $Proportion[,2]$ represents the secondary column of $Proportion$ matrix.

The direct copper input intensity of monetary flows to product sectors, matrix $\tilde{A}_{MP}$, and the intensity of inter-sectoral flow matrix among products that constitute physical copper output, matrix $\tilde{A}_{PP}$, are obtained as follows:

$$\tilde{A}_{MP} = \Gamma_{MP} \odot (\Phi_{MP} \cdot A_{MP})/Price_{2012} \tag{3}$$

$$\tilde{A}_{PP} = \Gamma_{PP} \odot (\Phi_{PP} \cdot A_{PP}) \tag{4}$$

Where Γ is the yield matrix in which the element (i,j) indicates the percentage of physical input i that is embedded in j and was estimated according to fabrication efficiency in literature; $\odot$ is the element-wise multiplication (Hadamard product); Φ is the mass filter which is a diagonal matrix with its diagonal elements being 0 or 1: element (i, i) of 1 indicates there is embedded physical copper output to other sectors in the monetary flows from sector i to other sectors.. Equation (4) and (5) are to remove the copper that will not be embedded as physically part of the output.

To allocate the copper from initial-stage manufacturers to final-stage manufacturers for sector j (Step 3 to Step 4 in Figure 1):

$$DirToFinal = \tilde{A}_{MP} \cdot diag((I - A_{PP})^{-1} \cdot diag(y\_P\_Tot)_{.j}\ ) \tag{5}$$

where the (i,j) element of $DirToFinal$ represents the copper semis from sector i after initial-manufacturing that is allocated to final-stage manufacturer sector j; $diag(y\_P\_Tot)_{.j}$ denotes the jth column of matrix $diag(y\_P\_Tot)$.

To calculate ML during final-stage manufacturing (ML2, Step4 to Step 5 in Figure 1) and NPC:

$$DirToFinal_{ML} = \tilde{A}_{MP} \cdot diag((I - (\Gamma_{PP} \odot A_{PP}))^{-1} \cdot diag(y\_P\_Tot)_{.j}) \quad (6)$$

$$DirToFinal_{ML\&NPC} = \tilde{A}_{MP} \cdot diag(\left(I - (\tilde{A}_{PP})\right)^{-1} \cdot diag(y\_P\_Tot)_{.j}) \quad (7)$$

where$DirToFinal_{ML}$ represents the copper semis embedded in final demand if only ML2 is considered; $DirToFinal_{ML\&NPC}$ represents the copper semis embedded in final demand as both ML2 and NPC are taken into accounted.

$$ML2 = DirToFinal - DirToFinal_{ML} \quad (8)$$

$$NPC = DirToFinal_{ML} - DirToFinal_{ML\&NPC} \quad (9)$$

The total embedded copper per unit product (in mass of copper semis per monetary value, denoted as specific copper use) is calculated as:

$$C = \tilde{A}_{MP} \cdot (I - \tilde{A}_{PP})^{-1} \quad (10)$$

where I is the identity matrix of the same size as $\tilde{A}_{PP}$.

The direct input intensity of copper from different sources into product sectors:

$$\tilde{A}_{MPs} = t(Proportion) \cdot diag(\tilde{A}_{MP}) \quad (11)$$

where the six rows of $\tilde{A}_{MPs}$ and $\tilde{A}_{MPs\_hypo}$ represent copper material from primary copper, New scrap, No.1 scrap, No.2 scrap, low-grade copper-bearing scrap and alloy scrap, respectively.

$$Cs = \tilde{A}_{MPs} \cdot (I - \tilde{A}_{PP})^{-1} \tag{12}$$

where Cs is the composition matrix that accounts for embedded copper "materials" from various sources in per unit products.

Copper use from different sources embedded in the final demand of different copper-embedded sectors:

$$CUs = Cs \cdot diag(y\_P\_Tot) \tag{13}$$

where the (i,j) element of CUs denotes the amount of copper semis from source i that is embedded in final demand for sector j.

Copper embedded in final demand in different demand categories:

$$FinalCate = diag(C) \cdot\ y\_P \tag{14}$$

where the (i,j) element of $FinalCate$ represents copper embedded in final demand for sector i by category j; y_P denotes the matrix of final demand by different categories.

To identify how each economic sector contributes to the specific copper use:

$$Contribution = \begin{pmatrix} diag(C) \cdot \tilde{A}_{PP} \\ \tilde{A}_{MP} \end{pmatrix} \tag{15}$$

Where the (i,j) element of $Contribution$ (excluding the last row) represents the amount of embedded copper in per unit sector j from sector i. It shows whether the specific copper use of sector j comes in the form of direct copper semis input (bottom row of $Contribution$ matrix) or as copper semis input that initially to other sectors but ended up in sector j. By normalizing the *Contribution* matrix by column sums, the portions contributed by each sector to the final demand of a certain sector is obtained.

APPENDIX S1-3. COPPER END-USE CATEGORIES

Researchers classify end-use categories in slightly different ways. In this paper, 408 economic sectors other than the 3 copper-related sectors in the 2012 USEEIO [4,5] were classified into 5 main end-use categories, further divided into 15 sub-categories adapted from Glöser et al.[3] (Table S1). The detailed end-use category assignment information for each sector was in Supporting Information 2 (SI2).

Table S1. End-use categories used in this paper (Adapted from Glöser et al.[3])

End-use categories	Sub-categories	Definition
Building construction	Plumbing	Water distribution, heating, gas, sprinklers
	Building plant	Air conditioning and tubes
	Architecture	Structures, roofs, gutters, flashing, decoration, builders
	Communications wire and cables	Wire and Cables
	Electrical power	Power distribution, earth, ground, light, wire device
Infrastructure	Power utility	Power transmission and distribution network

	Telecommunications	Telecommunication network
Industrial	Electrical industrial	Industrial transformer and motors
	Nonelectrical industrial	Valves, fittings, instruments and plant equipment
Transport	Automotive	Harnesses, motors, automotive electronics, radiators and tubing
	Other transport	Railroad, shipping and marine
Consumer and diverse	Consumer products	Appliances, instruments, tools and others
	Electronics	Industrial and commercial electronics and PCs
	Diverse	Services, ammunition, clothing, coins, and other
	Chemicals and powders	Chemicals and powders

APPENDIX S1-4. COPPER SCRAP TYPES

We used the pseudoclosed loop scrap allocation that assumes economic sectors only use their own scrap [7]. For example, miscellaneous unalloyed wire was recognized as No.2 copper scrap [8], thus the copper old scrap type that are used in the direct copper input to "Communication and energy wire and cable manufacturing" sector was set to be No.2 copper scrap. For machinery, copper alloy scrap was used[8]. For most sectors, we used the

copper content in the waste streams they generated [3,9] to decide the scrap type. Some professional judgement was also made. The detailed scrap type assignment information for each sector was in SI2.

APPENDIX S1-5. PROPORTION MATRIX

Due to the data limitation on EoL-RIR for different sectors, we used the coefficient of average EoL-RIR (0.19)/EoL-RR (0.45) [3] to obtain the Proportion matrix from the EoL-RR for each end-use category (Table S2).

Table S2. EoL-RR for each end-use category

End-use categories	Sub-categories	EoL-RR (Adapted from Glöser et al.[3])	EoL-RIR
Building construction	Plumbing	0.615	0.260
	Building plant	0.615	0.260
	Architecture	0.641	0.271
	Communications wire and cables	0.508	0.214
	Electrical power	0.579	0.244
Infrastructure	Power utility	0.534	0.225
	Telecommunications	0.508	0.214

Industrial	Electrical industrial	0.442	0.187
	Nonelectrical industrial	0.5	0.211
Transport	Automotive	0.49	0.207
	Other transport	0.469	0.198
Consumer and diverse	Consumer products	0.256	0.108
	Electronics	0.305	0.129
	Diverse	0.261	0.110
	Chemicals and powders	0	0

The detailed mass filter and yield matrix for all sectors were in SI2.

APPENDIX S1-6 MASS FILTER Φ AND YIELD MATRIX Γ

Mass filter was decided based on whether there is copper output from a certain sector. For example, the mass filter element of the sector "Scientific research and development services" was set to be 0 because there is no physical copper outflow from this sector to other sectors.

The material in this paper was already copper semis as illustrated in METHOD session. We identified the fabrication efficiency (processing yield) from copper semis to produce end-use product of each sector based on its end-use category (Table S3). The difference

of the two data sources are within the range of 0.1 except for electronics. We will use the average of these two sources as baseline value and set an uncertainty range of 0.1. Γ_{MP} was determined according to the assigned end-use categories of "Products". For Γ_{PP}, only those input sectors (rows) that were recognized by the authors as initial-stage users like wires and cables or dissipative users like chemicals were set to have a yield ratio based on their output (columns) end-use categories. Other sectors like motor components and residential buildings were assumed to have the yield ratio of 1 when they were performed as input sectors (rows).

Table S3. Yield for each end-use category

End-use categories	Sub-categories	Yield1(Adapted from Glöser et al.[3])	Yield2 (Adapted from Ruhrberg[9])	Average
Building construction	Plumbing	0.95	0.85	0.90
	Building plant	0.90	0.95	0.925
	Architecture	0.85	0.85	0.85
	Communications wire and cables	0.90	0.95	0.925
	Electrical power	0.90	0.90	0.90
Infrastructure	Power utility	0.85	0.85	0.85
	Telecommunications	0.90	0.90	0.90

Industrial	Electrical industrial	0.80	0.85	0.825
	Nonelectrical industrial	0.90	0.90	0.90
Transport	Automotive	0.825	0.875	0.85
	Other transport	0.8	0.875	0.838
Consumer and diverse	Consumer products	0.75	-	0.75
	Electronics	0.75	0.90	0.825
	Diverse	0.75	-	0.75
	Chemicals and powders	0.75	1	0.875

The detailed mass filter and yield matrix for all sectors were in SI2.

APPENDIX S1-7. COPPER PRICES

Copper 2012 price varied from 7,946 $/mt[10](London Metal Market, high-grade), to 7,962 $/metric ton[11] (grade A, minimum 99.9935% purity, cathodes and wire bar shapes, settlement price), to 8,100 $/metricton[10] (cathode). We used 8,100 as copper semis price in this paper as this price was specifically for the US producer.

APPENDIX S1-8. ENERGY CONSUMPTION OF COPPER SEMIS PRODUCTION IN THIS STUDY

For copper material from primary resources, energy use covers the copper production process from copper ore to semi-finished products. We first calculated Cumulative Energy Demand (CED) using Ecoinvent 3.6 database[12]. Primary copper cathode production had CED with an estimated range of 30~113 MJ/kg in various regions without considering the semis production and transport to market. By using the average CED of semis production ((7+10)/2 = 8.5 MJ/kg), the estimated range of primary copper semis production was 38.5~121.5 MJ/kg.

Among the different energy consumption values on primary copper cathode production in literature (Table S4**)**, this paper adopted the value of 57.3 MJ/kg as baseline value by assuming primary copper were produced from the copper ore grade of 0.5% based on the estimated world average copper ore grade 0.49% in 2010 [13]. In literature, depending on ore grades and technology used, energy consumption for primary copper cathode production could vary from 25.5 to 90 MJ/kg, therefore provide a range of 34~98.5 MJ/kg for primary copper semis production. Combining with the results calculated using Ecoinvent database, this paper used the value of 65.8 (57.3 + 8.5) MJ/kg as the baseline value for primary copper semis production with an uncertainty range of 34~121.5 MJ/kg.

Table S4. Energy consumption of primary copper cathode production in literature

Copper product form	Process (P-Pyrometallurgy or	Energy consumption (MJ/kg)	Note	Reference

	H-hydrometallurgy)			
Copper cathode	P	30	Mining, Concentration, Smelting, Refining (including sulfuric acid plant) or Services<br><br>Ore grade: 1.14%<br><br>Copper content in concentrates: 30%	[14]
		30-90	Cradle to gate	[15]
Copper cathode	P	16.9	From ore concentrate	[16]
Copper cathode	H	25.5	From oxide ores	[16]
Copper cathode	P	33	From ore 3% Cu	[17]
Copper cathode	P	57.3	From 0.5% Cu ore	[17]

Secondary copper production from scrap has been reported by Bureau of International Recycling (BIR)[16] as 6.3 MJ energy per kg of Cu without specifying whether the preparation process like collection were accounted for. For those from copper scrap, we consider energy use from scrap collection to semi-finished products. The results calculated from the Ecoinvent database do not differentiate among scrap of all qualities. Thus, we used the study from Kusik and Kenahan [18] (Table S5) that considered energy consumption of copper semis production from different grades of copper scrap including the process of scrap transportation, preparation, smelting, refining, melting, casting and pollution control and space heating where applicable. We further considered an uncertainty range from 90% ~ 110% of the current energy consumption. 90% of new scrap has high quality and is directly remelted and 10% is processed with EoL scrap through additional steps [3]. In this paper, the energy consumption of new scrap was assumed to be the same as No.1 scrap.

Table S5. Energy consumption of unit scrap input (Adapted from Kusik and Kenahan[18])

Scrap Type	MJ/metric ton
No.1/New scrap	4432
No.2	20089
Low-grade copper-bearing scrap	49345
Brass & Bronze scrap	8247

APPENDIX S1-9. Comparison of energy consumption in different studies

Table S6 Comparison of energy consumption in different studies

Primary refined copper (MJ/kg)	Secondary copper (MJ/kg)	Total energy consumption (PJ)	Share of primary production in total energy consumption	Region	Year	Reference
57.3 (25.5~113, depending on ore grades and technology used)	4.4~49.3 (semis, depending on scrap quality)	117	88%	US	2012	This study
82.6	23.8	536	88%	China	2010	[19]
28.4	4.9(direct melting)<br>22.9(smelting)	-	-	Europe	2010	[20]
100~150	8.4	About 2000	98%	Global	2010	[21]

The share of primary production in total energy consumption was particularly high in Elshkaki et al.[21] due to the low energy value used for secondary production, which also

indicates the importance of differentiating scrap quality when estimating energy consumption.

APPENDIX S1-10. POTENTIALLY RECYCLABLE RATES AND EOL-RR IN SCENARIO 2

Current EoL-RR are mostly well below the portions of potentially recyclable copper scrap in the total generated EoL copper scrap. Here we corresponded the end-use categories to those used in Ciacci et al. [22] and estimated the EoL-RR for all sectors under Scenario 2 by referring to the potentially recyclable rates. The correspondence relation was shown in Table S7. The detailed potentially recyclable rate for each economic sector was in SI2.

Table S7. Potentially recyclable rates for end-use categories

End-use category in this paper	Corresponding category in Ciacci et al.[22]	Potentially recyclable rate	EoL-RR in Scenario 2
Plumbing	Plumbing	0.98	1
Building plant/Cooling	Building plant	1	1
Architecture	Architecture	0.95	1
Communication	Communications	1	1
Electrical power	Electrical	1	1
Power utility	Electrical	1	1
Telecommunications	Communications	1	1
Electrical industrial	Industrial	1	1

Nonelectrical industrial	Industrial	1	1
Automotive	Transportation	0.99	1
Other transport	Transportation	0.99	1
Consumer and general products	Other-Miscellaneous	0.85	0.85
Electronics	Electronics	1	1
Diverse	Other-Miscellaneous	0.85	0.85
Chemicals and Powders	Other-Dissipative uses, Pigments & Chromated copper arsenate	0	0

APPENDIX S1-11. SCENARIO 3 EXPLANATION

Scenario 3 was to partly reflect the impact of technical coefficient and behavioral change on copper requirements. In the US, the total sales of vehicles in the year of 2012 was14779.5 thousand units with autos, light trucks and heavy trucks being 7245.2, 7188 and 346.3 thousand units, respectively. In 2019, the sales of autos, light truck and heavy trucks in the US were 4715, 12237.9 and 527.1 thousand units, respectively[23]. In 2019, the total copper consumption in the US was about 2486 kt[24] among which 20% was used in transportation equipment. In 2012, the copper used in transportation equipment accounted for about 17%[25]. By assuming that a fixed proportion of the copper used in transportation equipment was for vehicles in 2012 and 2019, copper used per unit of vehicles was increased by about 0.4% in the year of 2019 compared with that of 2012. In

order to mimic the economic structure situation of vehicles in 2019, in Scenario 3, wire and direct copper semis input into per unit output of motor electrical and electronic equipment and motor transmission and power train parts was increased by 0.4%, and the final demand for automobiles, light truck and heavy truck were about 65%, 170% and 152% of those in 2012, respectively.

APPENDIX S1-12. HEAT MAPS OF THE TOP FINAL DEMAND SECTORS IN TERMS OF TOTAL EMBEDDED COPPER AND THEIR CONTRIBUTORS FOR EACH FINAL DEMAND CATEGORY IN ALPHABETIC ORDER

Data used were provided in SI2.

In Figure S1-S15, X-axis represented the top sectors in decreasing order in certain category. Y-axis showed the major contributors of each top sector and the color in each cell showed the portion contributed by certain sector for the corresponding top demand sector as illustrated in the legend.

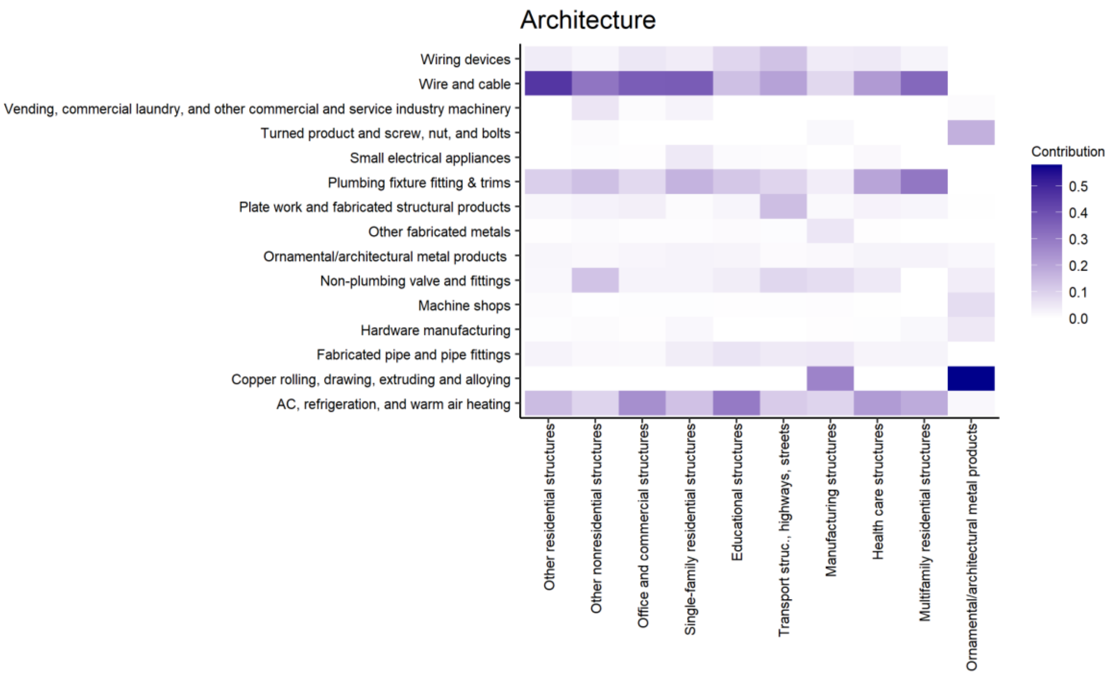

Figure S1. Contributions of various sectors to the top copper sectors in terms of total embedded copper for Architecture category

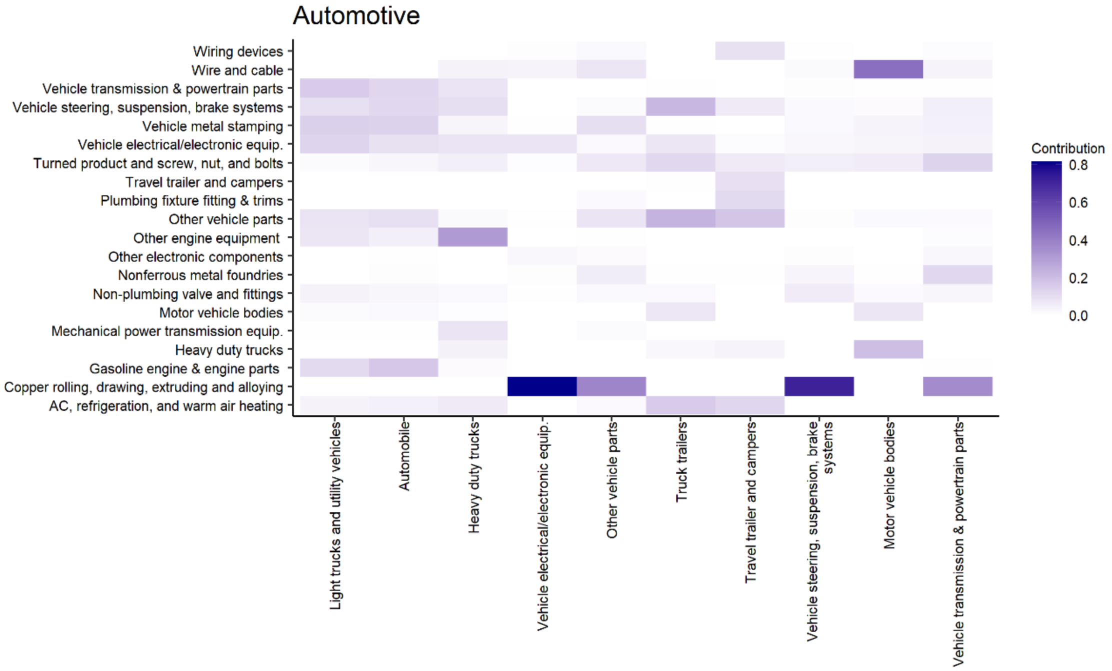

Figure S2. Contributions of various sectors to the top copper sectors in terms of total embedded copper for Automotive category

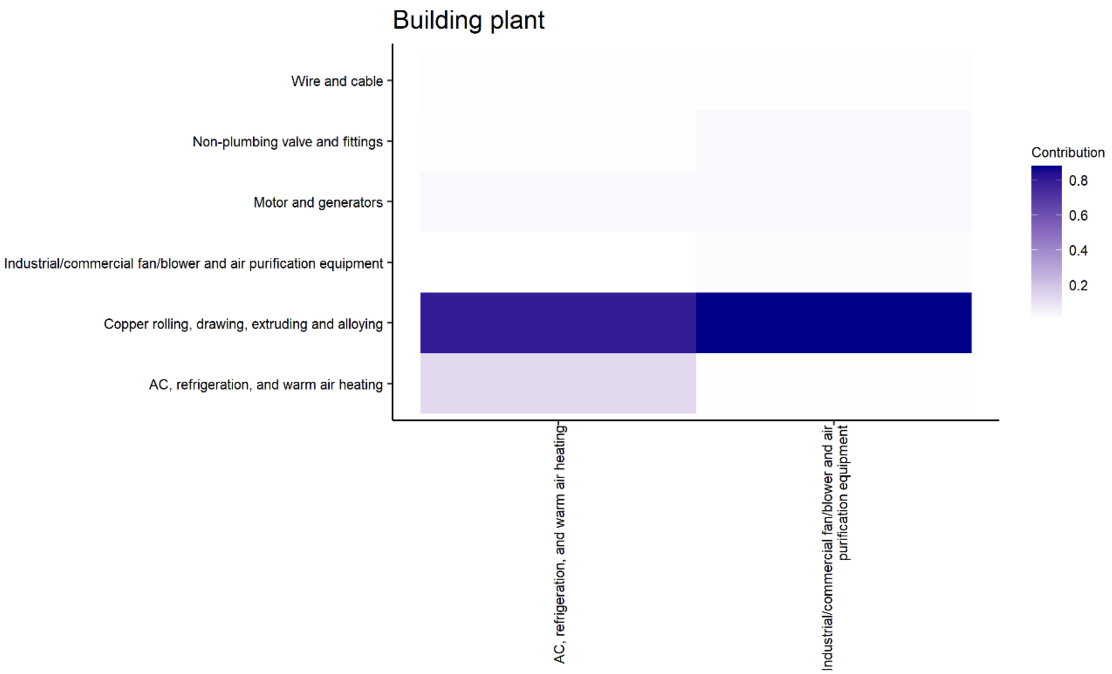

Figure S3. Contributions of various sectors to the top copper sectors in terms of total embedded copper for Building plant category

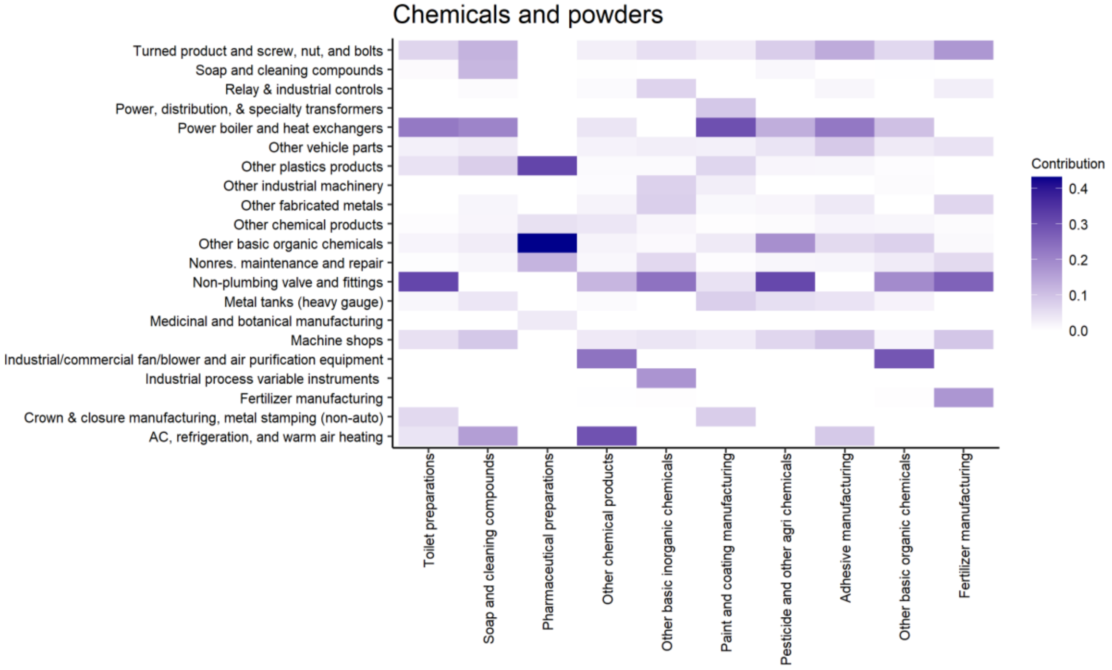

Figure S4. Contributions of various sectors to the top copper sectors in terms of total embedded copper for Chemicals and powders category

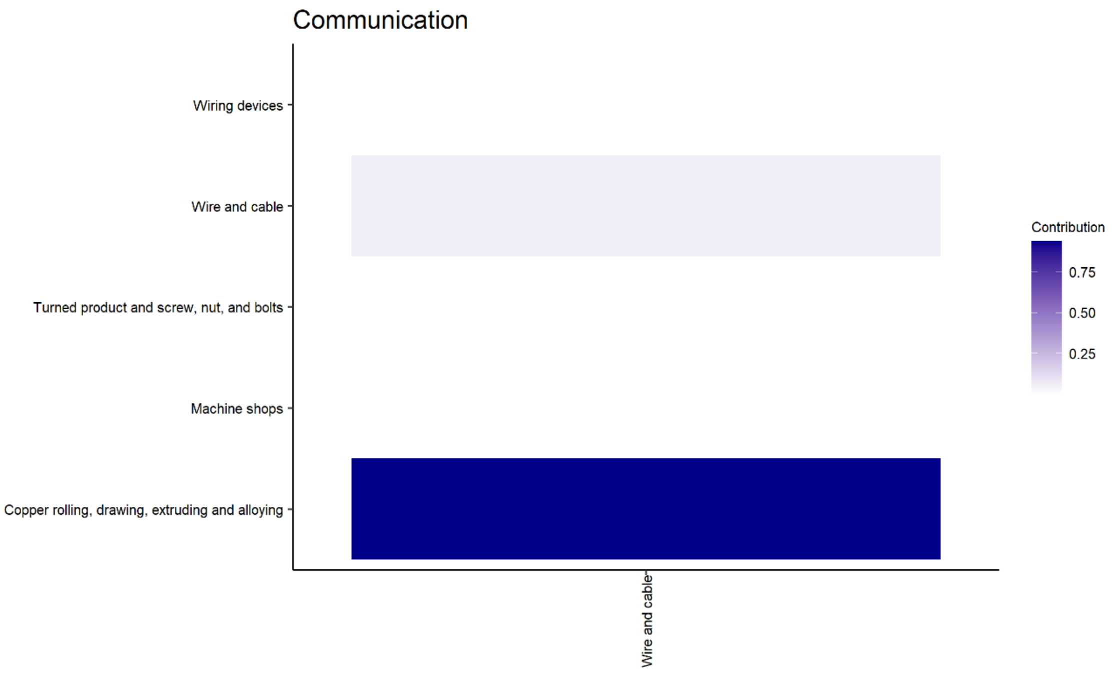

Figure S5. Contributions of various sectors to the top copper sectors in terms of total embedded copper for Communication category

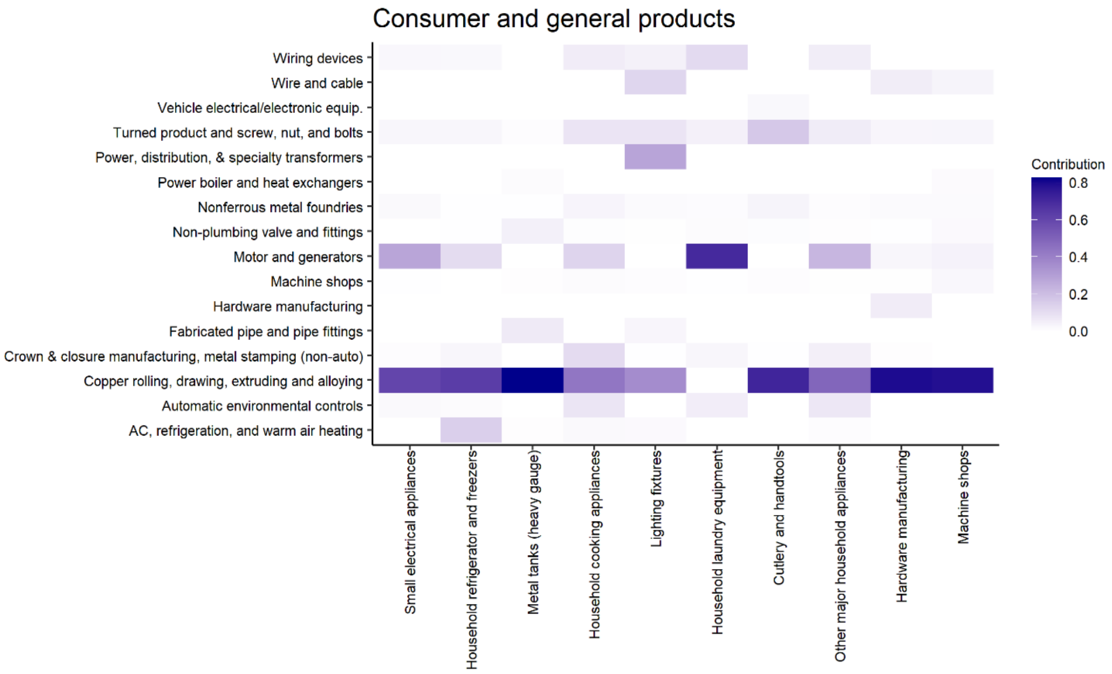

Figure S6. Contributions of various sectors to the top copper sectors in terms of total embedded copper for Consumer and general products category

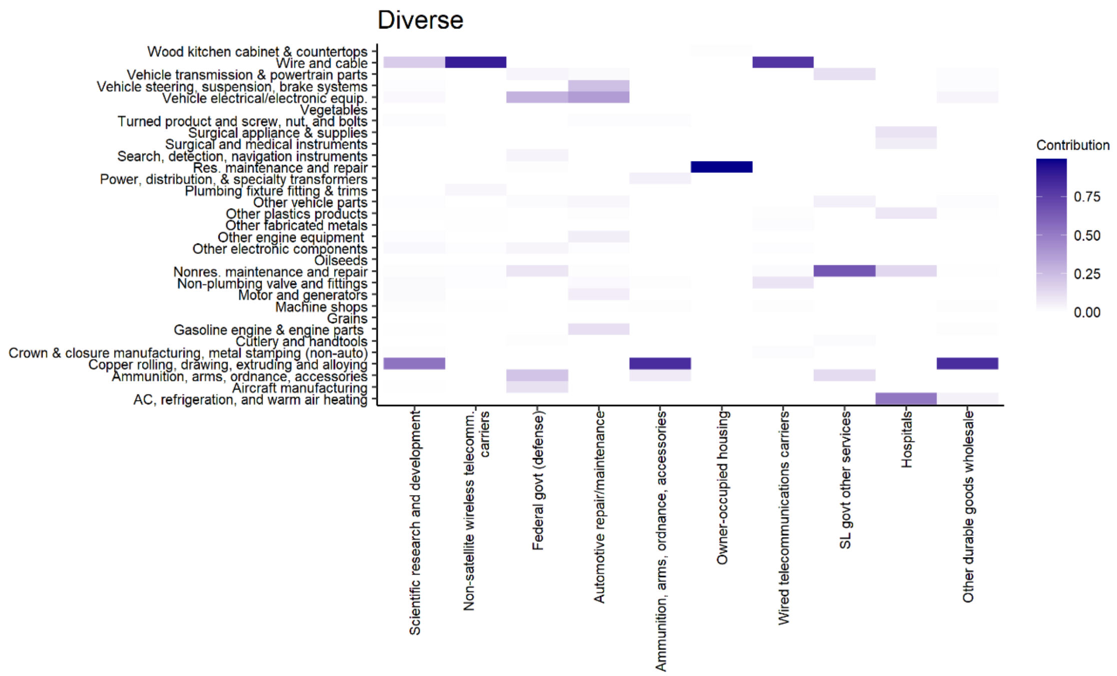

Figure S7. Contributions of various sectors to the top copper sectors in terms of total embedded copper for Diverse category

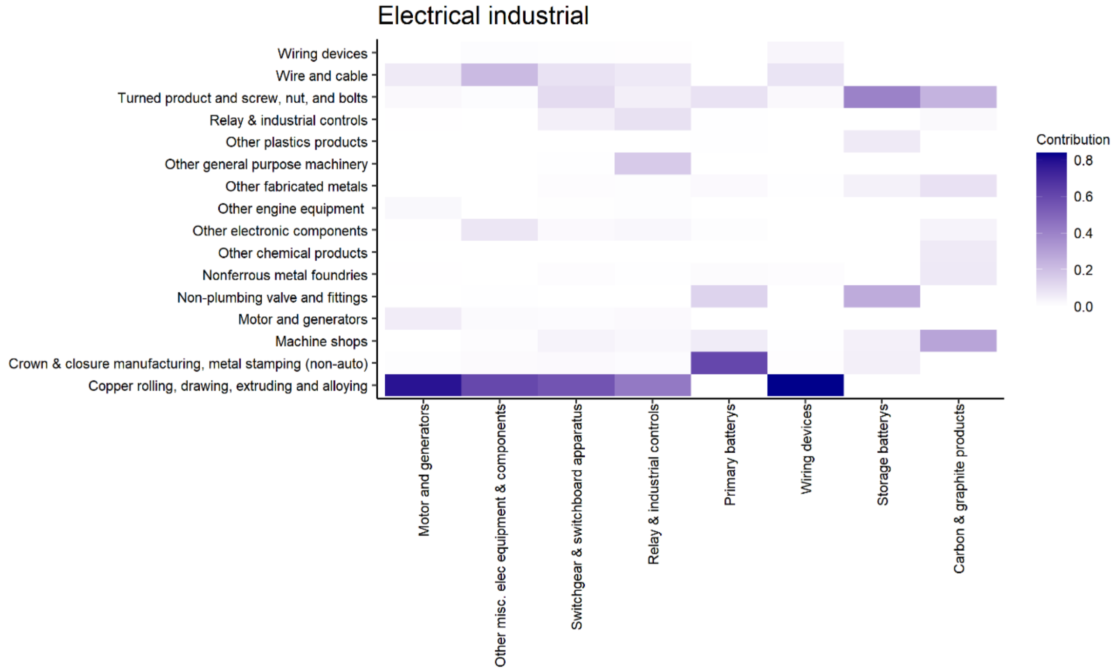

Figure S8. Contributions of various sectors to the top copper sectors in terms of total embedded copper for Electrical industrial category

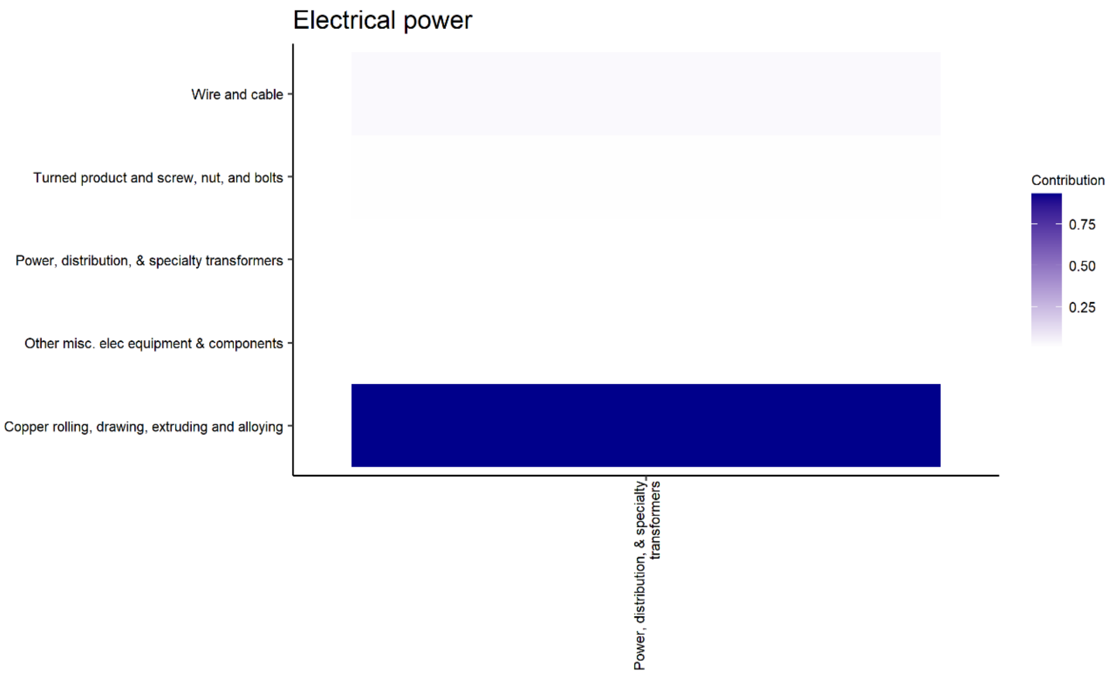

Figure S9. Contributions of various sectors to the top copper sectors in terms of total embedded copper for Electrical power category

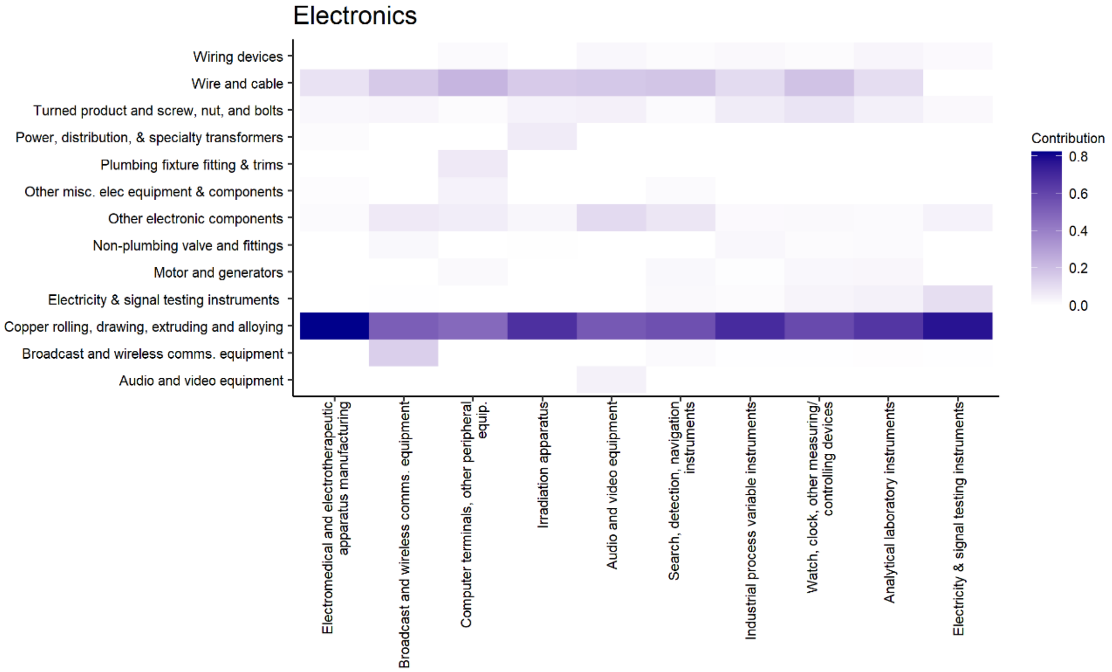

Figure S10. Contributions of various sectors to the top copper sectors in terms of total embedded copper for Electronics category

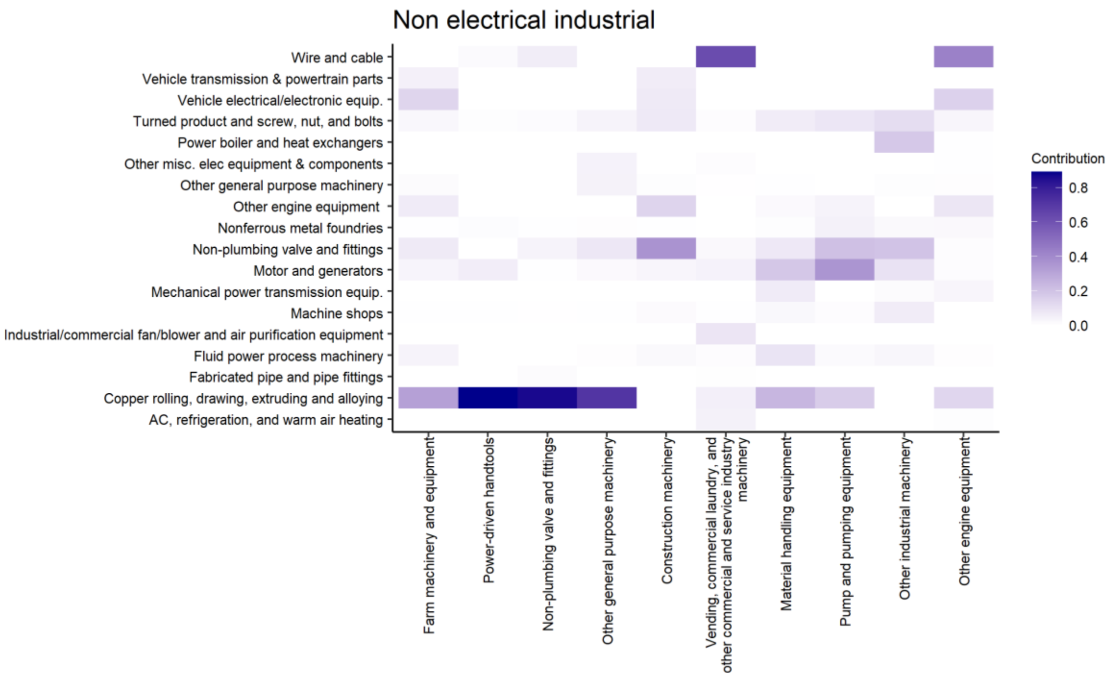

Figure S11. Contributions of various sectors to the top copper sectors in terms of total embedded copper for Non electrical industrial category

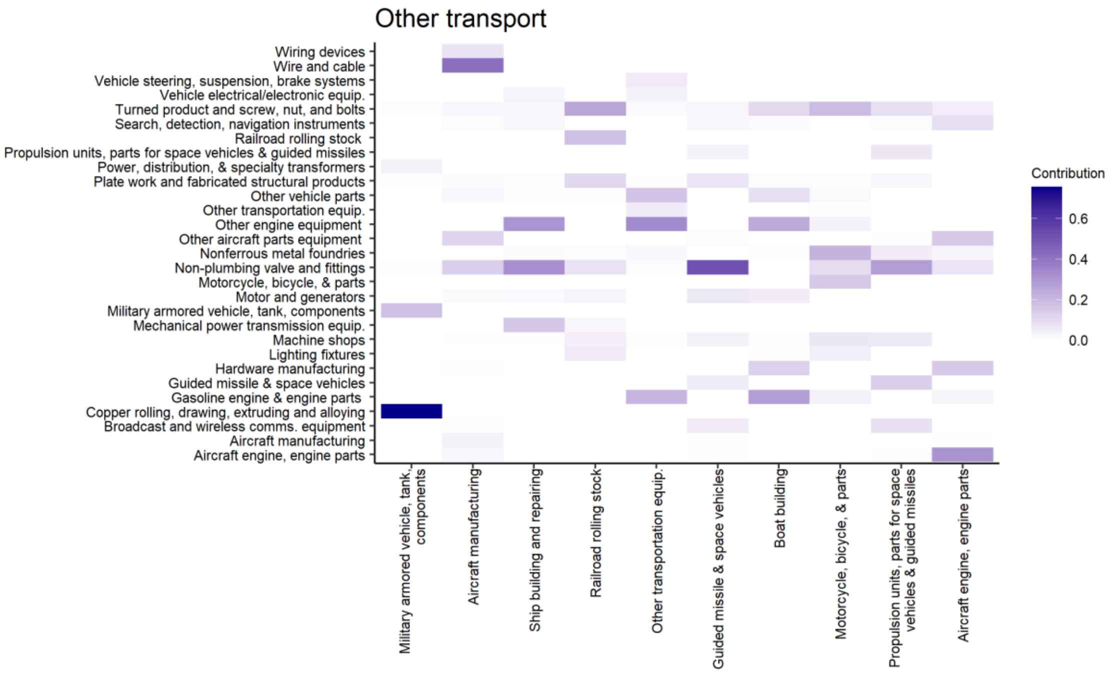

Figure S12 Contributions of various sectors to the top copper sectors in terms of total embedded copper for Other transport category

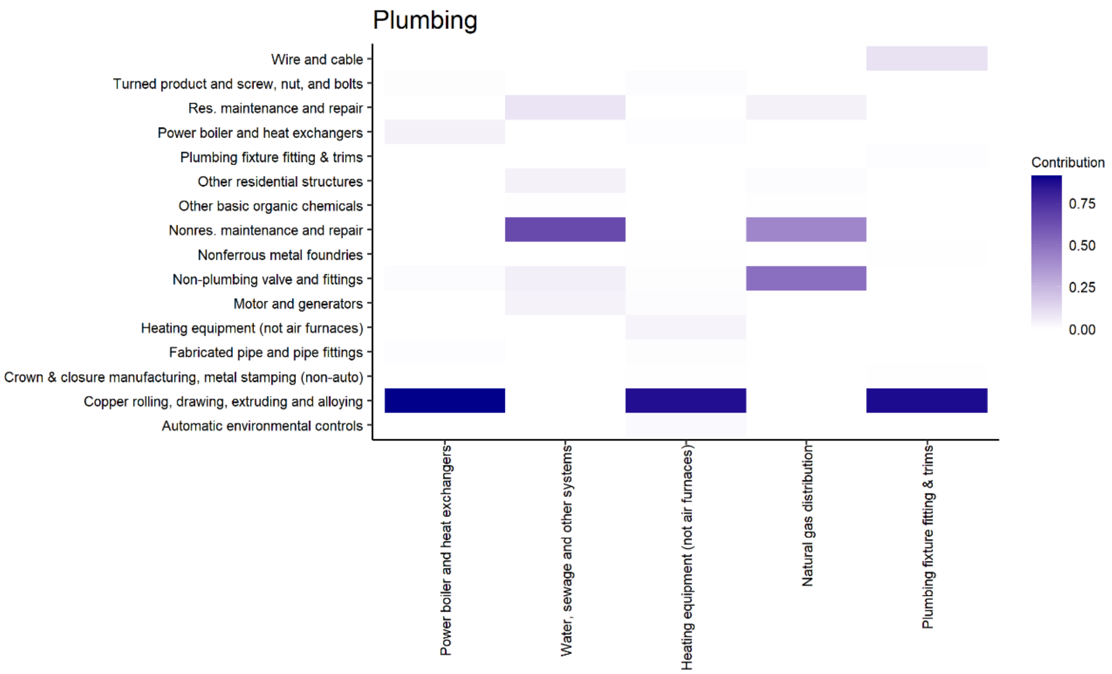

Figure S13. Contributions of various sectors to the top copper sectors in terms of total embedded copper for Plumbing category

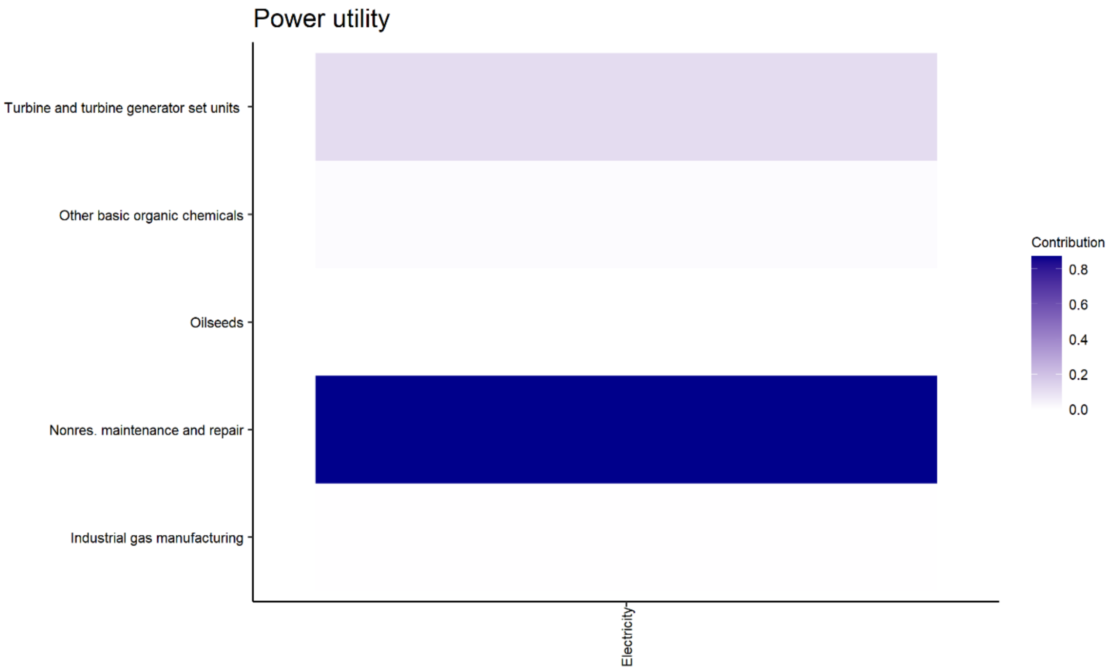

Figure S14. Contributions of various sectors to the top copper sectors in terms of total embedded copper for Power utility category

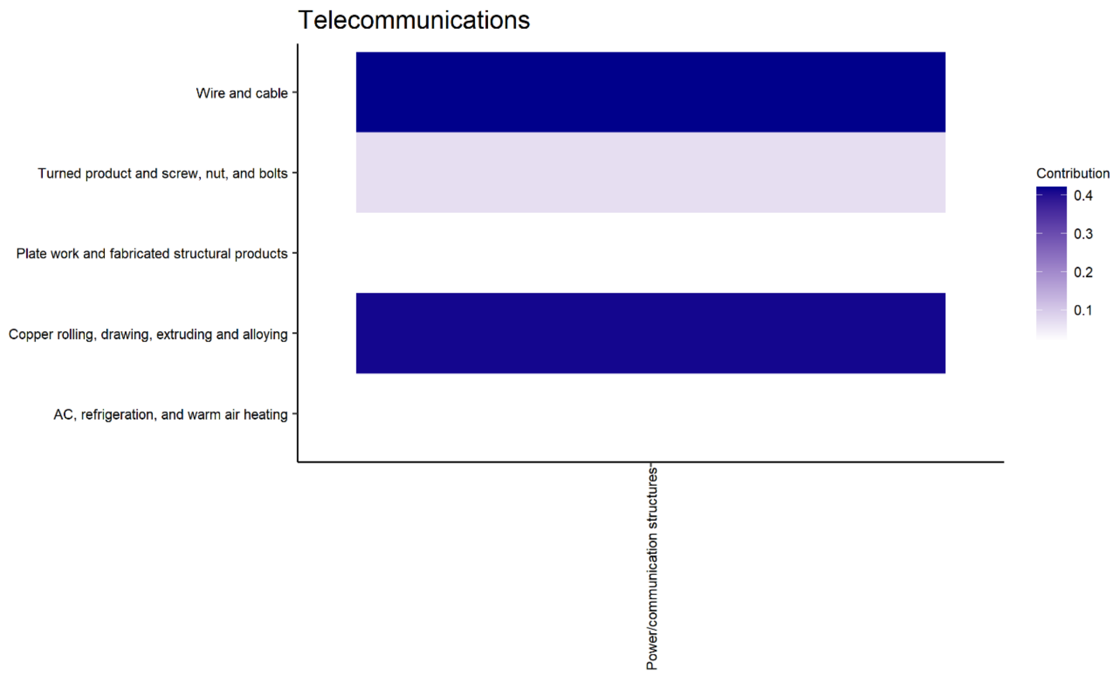

Figure S15. Contributions of various sectors to the top copper sectors in terms of total embedded copper for Telecommunications category

APPENDIX S1-13. PLAUSIBILITY CHECK OF EMBEDDED COPPER PER UNIT OF TWO AUTOMOTIVE SECTORS

Table S8. Embedded copper per unit of two automotive sectors

Sector	Specific copper-C from this model (kg/million USD)	Product current Price (USD/unit)	Embedded copper-C*Price in this model (kg/unit)	Literature (kg/unit)
Light truck	609	34,903[26]	21	20 [27]
Automobile manufacturing	532	21,900[28] ~26,701[26]	12-14	15~75 [27], 26[29]

APPENDIX S1-14. MARGINAL ENERGY SAVING

As shown in Figure S16, When EoL-RIR increased from current 20% to 41% in Scenario 2, marginal energy saving decreased as more low-grade scrap was recycled.

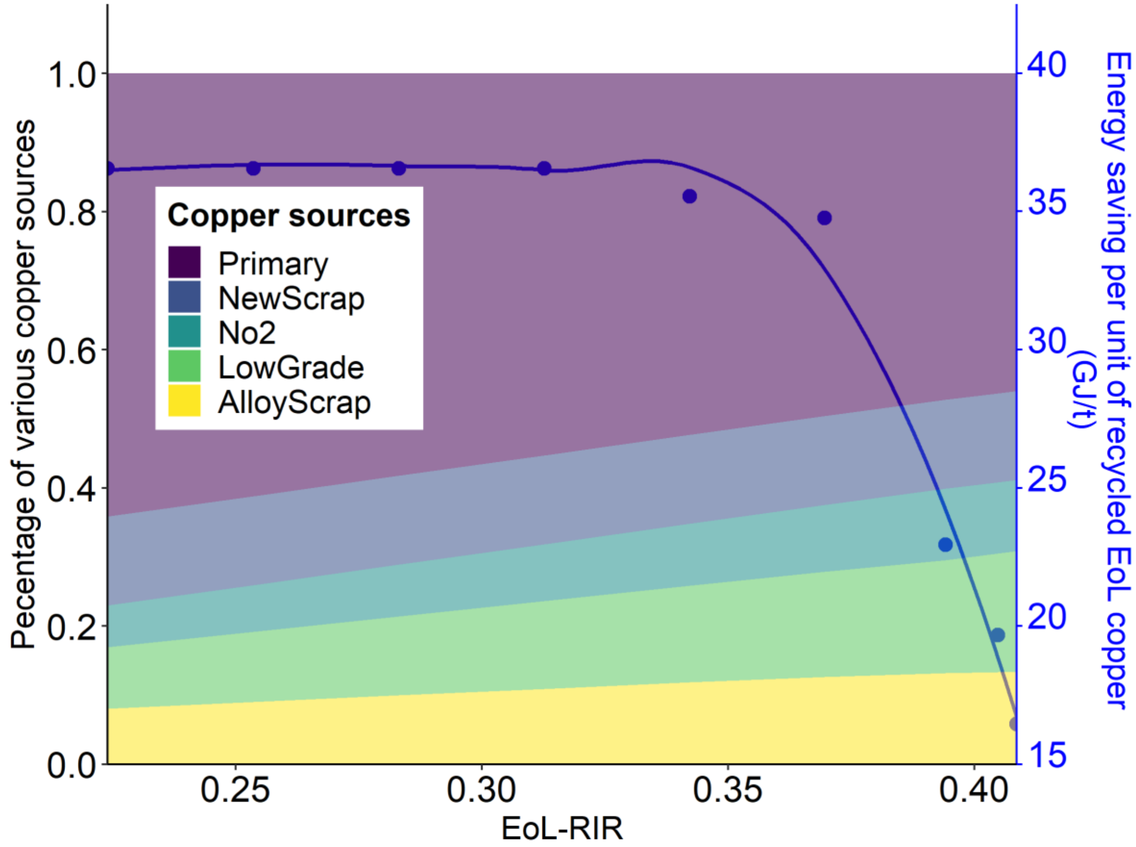

Figure S16 Marginal energy saving decreasing with EoL-RIR increasing

REFERENCES

(1) U.S. Geological Survey. Copper Statistics. In *Historical statistics for mineral and material commodities in the United States*; Kelly, T. D., Matos, G. R., Series Eds.; U.S. Geological Survey Data Series; 2014.

(2) Graedel, T. E.; Allwood, J.; Birat, J.-P.; Buchert, M.; Hagelüken, C.; Reck, B. K.; Sibley, S. F.; Sonnemann, G. What Do We Know About Metal Recycling Rates? *Journal of Industrial Ecology* **2011**, *15* (3), 355–366. https://doi.org/10.1111/j.1530-9290.2011.00342.x.

(3) Glöser, S.; Soulier, M.; Tercero Espinoza, L. A. Dynamic Analysis of Global Copper Flows. Global Stocks, Postconsumer Material Flows, Recycling Indicators, and Uncertainty Evaluation. *Environ. Sci. Technol.* **2013**, *47* (12), 6564–6572. https://doi.org/10.1021/es400069b.

(4) Berrill, P.; Miller, T. R.; Kondo, Y.; Hertwich, E. G. Capital in the American Carbon, Energy, and Material Footprint. *Journal of Industrial Ecology n/a* (n/a). https://doi.org/10.1111/jiec.12953.

(5) Miller, T. R.; Berrill, P.; Wolfram, P.; Wang, R.; Kim, Y.; Zheng, X.; Hertwich, E. G. Method for Endogenizing Capital in the United States Environmentally-Extended Input-Output Model. *Journal of Industrial Ecology* **2019**, *23* (6), 1410–1424. https://doi.org/10.1111/jiec.12931.

(6) NAICS Code: 331420 Copper Rolling, Drawing, Extruding, and Alloying https://www.naics.com/naics-code-description/?code=331420 (accessed Dec 12, 2019).

(7) Gaustad, G.; Olivetti, E.; Kirchain, R. Toward Sustainable Material Usage: Evaluating the Importance of Market Motivated Agency in Modeling Material Flows. *Environ. Sci. Technol.* **2011**, *45* (9), 4110–4117. https://doi.org/10.1021/es103508u.

(8) Samuelsson, C.; Björkman, B. Copper Recycling. In *Handbook of Recycling*; Elsevier, 2014; pp 85–94. https://doi.org/10.1016/B978-0-12-396459-5.00007-6.

(9) Ruhrberg, M. Assessing the Recycling Efficiency of Copper from End-of-Life Products in Western Europe. *Resources, Conservation and Recycling* **2006**, *48* (2), 141–165. https://doi.org/10.1016/j.resconrec.2006.01.003.

(10) U.S. Geological Survey. *Mineral Commodity Summaries 2013*; U.S. Geological Survey, 2013; p 198.

(11) Commodity Markets https://www.worldbank.org/en/research/commodity-markets (accessed Aug 31, 2020).

(12) ecoinvent https://www.ecoinvent.org/ (accessed Jan 4, 2020).

(13) Mudd, G. M.; Weng, Z.; Jowitt, S. M. A Detailed Assessment of Global Cu Resource Trends and Endowments. *Economic Geology* **2013**, *108* (5), 1163–1183. https://doi.org/10.2113/econgeo.108.5.1163.

(14) Alvarado, S.; Maldonado, P.; Barrios, A.; Jaques, I. Long Term Energy-Related Environmental Issues of Copper Production. *Energy* **2002**, *27* (2), 183–196. https://doi.org/10.1016/S0360-5442(01)00067-6.

(15) Voet, E. van der; United Nations Environment Programme; Working Group on the Global Metal Flows. *Environmental Risks and Challenges of Anthropogenic Metals Flows and Cycles. Report 3 Report 3*; 2013.

(16) Bureau of International Recycling (BIR). Report on the Environmental Benefits of Recycling. 2016.

(17) Bureau of International Recycling (BIR). Report on the Environmental Benefits of Recycling. 2008.

(18) Kusik, C. L.; Kenahan, C. B. *Energy Use Patterns for Metal Recycling*; Information circular 8781 - Bureau of Mines; U.S. Dept. of the Interior, Bureau of Mines: Washington DC, 1978.

(19) Dong, D.; van Oers, L.; Tukker, A.; van der Voet, E. Assessing the Future Environmental Impacts of Copper Production in China: Implications of the Energy Transition. *Journal of Cleaner Production* **2020**, *274*, 122825. https://doi.org/10.1016/j.jclepro.2020.122825.

(20) Ciacci, L.; Fishman, T.; Elshkaki, A.; Graedel, T. E.; Vassura, I.; Passarini, F. Exploring Future Copper Demand, Recycling and Associated Greenhouse Gas Emissions in the EU-28. *Global Environmental Change* **2020**, *63*, 102093. https://doi.org/10.1016/j.gloenvcha.2020.102093.

(21) Elshkaki, A.; Graedel, T. E.; Ciacci, L.; Reck, B. K. Copper Demand, Supply, and Associated Energy Use to 2050. *Global Environmental Change* **2016**, *39*, 305–315. https://doi.org/10.1016/j.gloenvcha.2016.06.006.

(22) Ciacci, L.; Reck, B. K.; Nassar, N. T.; Graedel, T. E. Lost by Design. *Environ. Sci. Technol.* **2015**, *49* (16), 9443–9451. https://doi.org/10.1021/es505515z.

(23) Gross Domestic Product | U.S. Bureau of Economic Analysis (BEA) https://www.bea.gov/data/gdp/gross-domestic-product#collapse86 (accessed Nov 7, 2020).

(24) U.S. Geological Survey. *Mineral Commodity Summaries 2020*; U.S. Geological Survey, 2020; p 200. https://doi.org/10.3133/mcs2020.

(25) U.S. Geological Survey. *Mineral Commodity Summaries 2014*; U.S. Geological Survey, 2014; p 196.

(26) Average price of new vehicles in the U.S. by type 1998-2016 https://www.statista.com/statistics/878742/new-vehicle-average-price-in-the-united-states-by-vehicle-type/ (accessed Jun 21, 2020).

(27) Dong, D.; Tukker, A.; Van der Voet, E. Modeling Copper Demand in China up to 2050: A Business-as-Usual Scenario Based on Dynamic Stock and Flow Analysis. *Journal of Industrial Ecology* **2019**, *23* (6), 1363–1380. https://doi.org/10.1111/jiec.12926.

(28) United Nations; Statistical Division. *2013 International Trade Statistics Yearbook*; 2014.

(29) Burnham, A. Updated Vehicle Specifications in the GREET Vehicle-Cycle Model. 40.
